# Lactoferrin-Decorated PLGA Nanoparticles for Targeted Tamoxifen Repurposing in Glioblastoma Cells

**DOI:** 10.3390/polym18091055

**Published:** 2026-04-27

**Authors:** Daniela Maria Sousa, Maria João Ramalho, Stéphanie Andrade, Joana Angélica Loureiro, Jorge Lima, Maria Carmo Pereira

**Affiliations:** 1 LEPABE—Laboratory for Process Engineering, Environment, Biotechnology and Energy, ALiCE—Associate Laboratory in Chemical Engineering, Faculty of Engineering, University of Porto, Rua Dr. Roberto Frias, 4200-465 Porto, Portugal; 2 Department of Mechanical Engineering, Faculty of Engineering, University of Porto, Rua Dr. Roberto Frias, 4200-465 Porto, Portugal; 3 i3S—Institute for Research and Innovation in Health, University of Porto, R. Alfredo Allen, 4200-135 Porto, Portugal; 4 Ipatimup—Institute of Molecular Pathology and Immunology, University of Porto, Rua Júlio Amaral de Carvalho 45, 4200-135 Porto, Portugal; 5 Faculty of Medicine, University of Porto, Alameda Prof. Hernâni Monteiro, 4200-319 Porto, Portugal

**Keywords:** nanomedicine, brain delivery, brain tumor, drug resistance, MGMT-mediated resistance, non-alkylating drug

## Abstract

Glioblastoma (GB) classical treatment with the alkylating drug temozolomide (TMZ) is not effective mainly due to chemoresistance mechanisms, particularly those mediated by O6-methylguanine-DNA methyltransferase (MGMT). In this context, polyethylene glycol (PEG)-coated poly(lactic-co-glycolic acid) (PLGA) nanoparticles (NPs) were developed to deliver tamoxifen (TAX), a clinically approved non-alkylating drug with reported anti-GB activity. The NP formulation was optimized using a factorial design and subsequently functionalized with lactoferrin (Lf) to enhance GB targeting. The Lf-conjugated optimized formulation exhibited a mean diameter of 193 ± 6 nm, a polydispersity index (PDI) of 0.11 ± 0.04, a zeta potential of −18.2 ± 6.8 mV, and an encapsulation efficiency (EE) of 68.6 ± 1.8%. The NPs exhibited a sustained release profile for up to 23 days, and remained stable under physiological conditions. Cell uptake studies, conducted in human GB cells (U87, U251, and T98G) and healthy astrocytes, demonstrated enhanced internalization of Lf-NPs in GB cells compared with non-conjugated NPs, suggesting uptake through Lf-binding site-mediated endocytosis. Cytotoxicity assays further indicated that Lf-conjugation improved the antiproliferative efficacy of TAX-loaded NPs relative to non-functionalized formulations, particularly in GB cells. Moreover, combination studies with TMZ showed that the developed NPs were able to sensitize GB cells to treatment with this alkylating agent. In sum, this work supports the potential of the developed Lf-decorated TAX-loaded PLGA NPs as a nanoplatform for targeted delivery against GB.

## 1. Introduction

Glioblastoma (GB) is a highly aggressive brain tumor arising from astrocytic lineage cells and represents the most severe form of diffuse glioma, classified as grade IV by the World Health Organization (WHO). It is marked by rapid tumor progression and extensive infiltration into surrounding brain tissue. GB accounts for more than half of all gliomas and remains the most frequently diagnosed malignant primary brain tumor [1]. Its current therapeutic approach, combining surgical resection with radiotherapy and temozolomide (TMZ)-based chemotherapy, leads to only modest improvements in patient survival. In fact, the prognosis remains poor, with median survival typically not exceeding 15 months [2].

TMZ treatment is associated with several adverse effects, ranging from common symptoms such as nausea, fatigue, and hair loss to more severe hematological complications, including leukopenia, neutropenia, and thrombocytopenia [3]. Furthermore, resistance to TMZ is a major clinical challenge. Around 50% of GB patients show limited or no response to therapy, largely due to elevated levels of O6-methylguanine methyltransferase (MGMT), a DNA repair enzyme that counteracts TMZ-induced damage [4].

Thus, in this work, tamoxifen (TAX), a selective estrogen receptor modulator clinically approved for breast cancer therapy, is proposed as part of a drug repositioning strategy, which explores new therapeutic applications for existing pharmaceutical agents [5]. Unlike traditional chemotherapeutics, such as TMZ, TAX is a non-alkylating agent, meaning its efficacy is not compromised by MGMT-mediated resistance. TAX functions through multiple pathways, including the inhibition of protein kinase C (PKC), which is a pathway often active in aggressive brain tumors, including GB, as PKC is frequently overexpressed or hyperactivated in central nervous system (CNS) tumors [6,7]. PKC is a key regulator of multiple cellular processes involved in cell proliferation and transformation. By inhibiting this pathway, TAX disrupts signaling mechanisms that drive cell cycle progression and tumor growth [8].

Despite TAX’s potential to treat GB, it still faces challenges that limit its effectiveness, including poor water solubility, limited tissue selectivity, and the difficulty of maintaining consistent therapeutic concentrations in tumor tissues due to its physicochemical properties [9,10]. Rather than exploring novel PKC inhibitors, a drug repositioning strategy was selected for this work, as it allows the use of a clinically established compound with known safety. To overcome its current limitations and improve its therapeutic efficacy, TAX can be encapsulated in nanoparticles (NPs), thereby enhancing its ability to achieve and sustain effective concentrations in tumor tissues [10,11]. Modifying NPs’ surfaces with hydrophilic polymers, such as polyethylene glycol (PEG), enables them to evade immune recognition and remain in circulation longer, increasing the likelihood that NPs reach brain tissue [12].

Nevertheless, drug delivery to brain tumors still faces an additional challenge, crossing the blood–brain barrier (BBB). A widely used approach to overcome this obstacle is to decorate the NPs’ surface with targeting ligands that bind receptors overexpressed on BBB endothelial cells and in GB cells. An example of this strategy is lactoferrin (Lf), which can interact with receptors such as the low-density lipoprotein receptor-related protein 1 (LRP1, also known as CD91), reported to be overexpressed in both BBB endothelial cells and GB cells. For this reason, Lf is a suitable targeting ligand for GB [13,14].

Therefore, this work aimed to develop Lf-functionalized PEG-coated poly(lactic-co-glycolic acid) (PLGA) NPs encapsulating TAX for targeted delivery to GB cells. PLGA is a copolymer widely used in the formulation of biomedical drug-delivery systems, owing to its biodegradability, proven safety, and biocompatibility, as well as its versatility for both formulation and surface functionalization [15]. Most published studies involving TAX-loaded nanoformulations focus on breast cancer applications [9,16,17]. To date, only a few studies have been published on TAX and NPs for GB application, and most strategies developed have used TAX as a functional moiety at the NPs’ surface rather than incorporating it as the active drug within the delivery system [18,19,20,21]. Based on the current literature, Lf-conjugated PLGA NPs have not yet been reported for TAX delivery in the context of GB therapy.

NP production was optimized by implementing a factorial design, and the nanocarriers were surface-decorated with Lf to improve targeting specificity toward GB cells. Following optimization, the NPs’ physicochemical properties, morphology, colloidal stability, and drug-release kinetics were studied in vitro. To evaluate their in vitro biological performance, uptake studies were conducted in three human GB cell lines (U87, U251, and T98G) and healthy human astrocytes to evaluate the uptake of Lf-functionalized NPs and the involvement of Lf-binding sites via competitive blocking assays. Furthermore, the anti-GB activity of TAX-loaded NPs was evaluated and compared with free TAX, as well as their ability to sensitize GB cells to TMZ therapy.

## 2. Materials and Methods

### 2.1. Materials

Polyvinyl alcohol 4-88 (Mowiol^®^ 4-88, MW 31,000, 88% hydrolyzed) (PVA), PLGA Resomer^®^ RG 503 H (50:50; MW 24,000–38,000), 1-ethyl-3-(3-dimethylaminopropyl) cardodiimide (EDC) (MW 191.70, purity ≥ 98%), dichloromethane (DCM) (MW 84.93, 99.8%), tamoxifen citrate (MW 371.51, purity ≥ 98%), methoxy polyethylene glycol amine (mPEG-NH_2_) (MW 2.000, purity ≥ 99%), recombinant human Lf (MW ≈ 80 kDa, purity > 90%), coumarin-6 (C6, MW 350, purity ≥ 98%), phosphate-buffered saline (PBS) tablets (OmniPur^®^, Calbiochem^®^), dimethyl sulfoxide (DMSO) (MW 78.13, ≥99.9%,), trypsin solution from porcine pancreas, acetic acid (MW 60.05, purity ≥ 98%), trichloroacetic acid (TCA) (MW 163.38, purity ≥ 99%), sulforhodamine B (SRB) (MW 580.66), tris(hydroxymethyl)aminomethane (MW 141.14, purity ≥ 99.8%), sodium hydroxide (NaOH, MW 40.0, purity ≥ 97%) and polyoxyethylene (10) isooctylcyclohexyl ether (Triton X-100) were purchased from Sigma Aldrich (St. Louis, MO, USA). Ethyl acetate (EA) (MW 88.11, purity 99.6%, ACS reagent) was acquired from BioVision (Waltham, MA, EUA). The Pierce™ BCA Protein Assay Kit was acquired from Thermo Fisher Scientific (Waltham, MA, USA). Diethyl ether (MW 74.12, ACS Reagent, purity ≥ 99.8%) was obtained from Honeywell Riedel-de Haën (Seelze, Germany), and methanol (HiPerSolv CHROMANORM^®^, MW 32.04, purity ≥ 99.9%) was purchased from VWR International (Rosny-sous-Bois, France). Uranyl acetate dihydrate (MW 424, purity > 99%) was obtained from GenScript Biotech (Piscataway, NJ, USA). TMZ (MW 194.15, purity ≥ 99%) was acquired from Selleck Chemicals (Munich, Germany). Fungizone (Amphotericin B Solution, 100×), Penicillin-streptomycin (Pen-Step) solution (×100), and Dulbecco’s Modified Eagle Medium (DMEM) high Glucose (4.5 g/L) with stable glutamine were obtained from Capricorn Scientific GmbH (Ebsdorfergrund, Germany). Fetal bovine serum (FBS), heat-inactivated, was purchased from Biowest LCC (Riverside, MO, USA). Trypan blue (MW 960.8, dye content > 70%) was obtained from Biochem Chemopharma (Cosne Sur Loire, France). All aqueous solutions were prepared in deionized, filtered, ultrapure Milli-Q water (Milli-Q SQ 2 Series, Millipore, France). PBS solutions were prepared in ultrapure water and filtered before use. All remaining reagents were used as received. Reagents used for cell culture and NP preparation were sterilized by autoclaving when appropriate.

### 2.2. Cell Lines and Culture Conditions

Human GB cell lines (U87, U251, and T98G) and human healthy immortalized astrocytes (NHA) were used in this study. The cells were purchased from the American Type Culture Collection (ATCC^®^, Manassas, VA, USA). The GB cell lines were used as different representative in vitro models of GB, whereas NHA cells were used as a non-tumoral control. All cells were grown in DMEM supplemented with 10% (*v*/*v*) FBS, 1% (*v*/*v*) Pen-Strep, and 0.5% (*v*/*v*) fungizone to prevent microbial contamination. Cell cultures were maintained at 37 °C in a humidified incubator with 5% CO_2_. Cells were routinely monitored and passaged upon reaching approximately 80% confluence. For subculturing, cells were detached using trypsin and reseeded according to the requirements of each experimental assay.

### 2.3. Optimization of the Protocol for TAX-Loaded PEG-PLGA NPs Preparation

#### 2.3.1. Synthesis of PEG–PLGA Conjugate

An EDC-mediated coupling reaction was used to achieve PEG-PLGA conjugation in this work. Initially, 2.0 mg of PLGA was dissolved in 200 µL of DCM, to which EDC was added in a ten-fold molar excess relative to PLGA. The solution was stirred at room temperature for 1 h to activate the carboxyl groups of PLGA (100 rpm), facilitating the subsequent reaction with PEG [22]. Following the activation step, a 200 µL DCM solution containing 1.0 mg of mPEG-NH_2_ was added to the activated PLGA mixture, and the reaction was stirred overnight at 250 rpm (revolutions per minute) using a microplate shaker (VWR, Radnor, PA, USA) to promote efficient conjugation. The PLGA-PEG conjugate was collected by precipitation with a cold mixture of diethyl ether and methanol (70:30 *v*/*v* ratio) [23]. The mixture was then briefly stirred and centrifuged at 14,500 rpm for 30 min (MiniSpin Plus, model 5453, Eppendorf AG, Hamburg, Germany). The obtained pellet was resuspended in EA. Under these conditions, unreacted EDC and its urea byproduct are expected to remain in the supernatant due to their greater solubility and to be removed during the purification step.

#### 2.3.2. Production of TAX-Loaded PLGA NPs

NPs were produced by the single emulsion evaporation of solvent method, which is well-suited for the encapsulation of hydrophobic molecules such as TAX [24]. Initially, a polymer solution was prepared by dissolving a known amount of PLGA together with 1.0 mg of the previously synthesized PEG–PLGA conjugate, and 1.0 mg of TAX in a known volume of EA. The quantities of EA and PLGA varied among the different formulations, as described in Table 1 in Section 2.3.3. To generate an oil-in-water emulsion, a known volume of PVA solution with a known concentration was added to the organic phase. The PVA volume and concentration varied among the different formulations, as described in Table 1 in Section 2.3.3. Then, the mixture was vortexed (30 s) to promote emulsification and was then exposed to ultrasonic homogenization using a probe sonicator (UP400S, Hielscher, Berlin, Germany) at 70% amplitude. The duration of the sonication cycles varied across the different formulations, as described in Table 1 in Section 2.3.3. To prevent overheating during sonication, the container was immersed in an ice bath.

Immediately following sonication, the emulsion was placed in a glass beaker and stirred at 800 rpm for 1 h on a magnetic stirring plate (electromagnetic stirrer, Colorsquid, ika^®^, Staufen, Germany) to facilitate complete evaporation of the organic solvent. The resulting NPs were collected by centrifugation, employing gradually increasing speeds (10,000 to 14,500 rpm) and durations (2 to 15 min). The supernatant was collected to further quantify the non-entrapped drug. The pellet containing the NPs was resuspended in ultrapure water (1.0 mL) and saved for future physicochemical characterization.

#### 2.3.3. Design of Experiments-Factorial Design

NP production was optimized using a full factorial design, allowing for the evaluation of the effects of four independent variables at two levels: low (−1) and high (+1). The independent variables selected for optimization, based on our preliminary studies, were the amount of PLGA (mg), the number of sonication cycles, the PVA concentration (% *w*/*v*), and the volume ratio between EA and the aqueous PVA phase.

A total of 35 experimental runs were generated, in which the center point (0) was replicated three times to assess experimental reproducibility, while all other combinations were prepared in duplicate to ensure statistical robustness and improve model fitting. Table 1 summarizes the variables and the levels applied in the design. In this context, the coded values −1, 0, and +1 represent the lowest, intermediate, and highest experimental levels of each variable, respectively.

The response variables considered in the optimization were particle size, polydispersity index (PDI), zeta potential, encapsulation efficiency (EE), and loading capacity (LC). The optimization goals were defined as follows: particle size was targeted at 120–200 nm, PDI was minimized (target value below 0.2), zeta potential was minimized, and both EE and LC were maximized, with a minimum EE threshold of 40%.

The factorial design and corresponding experimental plan were generated with the Design-Expert^®^ software (version 13, Stat-Ease Inc., Minneapolis, MN, USA). The design was randomized to avoid systematic bias. This platform also enabled statistical analysis and model generation for each response variable. The experimental data obtained were analyzed using analysis of variance (ANOVA), which evaluated the significance of the individual factors and their interactions based on F-values and associated *p*-values. A confidence level of 95% (*p* < 0.05) was adopted to determine statistical significance. Model adequacy was further assessed using diagnostic plots (normal probability plots, and residuals versus predicted values) and regression coefficients (R^2^).

### 2.4. Surface Modification of TAX-Loaded PLGA NPs with Lf

The surface decoration of TAX-loaded PLGA NPs was performed using Lf as a targeting ligand. To this end, the optimized NP formulation was prepared following the protocol detailed in Section 2.3.2, but employing the optimal parameters identified through the DOE approach. Following centrifugation, the pellet containing 50 mg of PLGA NPs was resuspended in ultrapure water (1.0 mL) and the resulting NPs were then conjugated with Lf.

To activate the carboxylic groups on the PLGA surface, EDC was added in a ten-fold molar excess relative to PLGA (284.8 µL of an aqueous EDC solution at a concentration of 10 mg/mL), and the mixture was stirred at room temperature (30 min, 100 rpm). Subsequently, 207.8 µL of an aqueous Lf solution at 1 mg/mL was added to the activated NPs and incubated under agitation at room temperature (100 rpm) for 1 h. Lf was added in a two-fold molar excess relative to the amount required to achieve an average of approximately 100 Lf molecules per NP. This molar excess was determined using particle number estimates based on NP mass, average size, PLGA density, following standard assumptions of spherical geometry. The total number of NPs in the suspension was first estimated, and based on this, the theoretical number of Lf molecules required to reach the desired surface density was calculated.

After conjugation, NPs were recollected by centrifugation as detailed in Section 2.3.2, resuspended in 1.0 mL of ultrapure water, and saved for further physicochemical characterization.

The unbound Lf present in the supernatant was quantified using the Pierce™ BCA Protein Assay Kit, based on the bicinchoninic acid (BCA) reaction using a microplate reader (Synergy 2 Microplate Reader, BioTek, Swindon, UK), in which peptide bonds in an alkaline medium reduce Cu^2+^ to Cu^+^ via the biuret reaction. The generated Cu^+^ then chelates with two molecules of BCA to form a soluble, purple-colored complex whose absorbance at 562 nm can be correlated with Lf concentration [25].

### 2.5. NPs Physicochemical Characterization

#### 2.5.1. Dynamic Light Scattering Coupled with Laser Doppler Velocimetry

The NPs’ physicochemical characterization was performed using dynamic light scattering (DLS) coupled with laser Doppler velocimetry (LDV) using a ZetaSizer Nano ZS (Malvern Instruments, Malvern, UK). These techniques were employed to determine the mean hydrodynamic size, PDI, and zeta potential of the NPs. For the measurements, the NP suspension was diluted to obtain a working concentration of 1.0 mg/mL. A standard polystyrene cell (Sarstedt^®^) and a DST1070 cell (Malvern Panalytical) were used for DLS and LDV measurements, respectively. The water’s refractive index, used as the dispersant at 25 °C, was set to 1.330, while its dielectric constant and viscosity were set to 78.5 and 0.8872 cP, respectively. The PLGA refractive index was fixed at 1.59, with an absorption value of 0.01.

DLS and LDV measurements were performed after NPs’ production and over time to assess the colloidal stability of the NPs in storage conditions by monitoring changes in zeta potential, size and PDI. For this purpose, the NPs were stored as an aqueous suspension at 4 °C, and DLS/LDV measurements were performed weekly over approximately 5 weeks (36 days).

#### 2.5.2. Determination of the TAX Encapsulation Efficiency and NPs’ Loading Capacity

The non-encapsulated TAX was determined by UV-Vis spectrophotometry using a microplate reader (Synergy 2 Microplate Reader, BioTek, UK). Absorbance values were measured and interpolated using a previously established TAX calibration curve in ultrapure water. The EE and LC were then determined using the following equations [26]:

EE (%) = (Total amount of TAX (mg) − non encapsulated TAX (mg))/(Total amount of TAX (mg)) × 100,(1)

LC (%) = (Total amount of TAX (mg) − non encapsulated TAX (mg))/(PLGA (mg)) × 100.(2)

#### 2.5.3. Attenuated Total Reflectance Fourier Transform Infrared Spectroscopy

The presence of Lf on the surface of the NPs was confirmed by attenuated total reflectance (ATR) Fourier Transform Infrared Spectroscopy (FTIR). A Bruker Alpha FTIR spectrometer (Alpha, Bruker Optik GmbH, Ettlingen, Germany) and Opus Software (version 6.5, Bruker Optik GmbH) were used to record the ATR-FTIR spectra of the following samples: TAX-loaded PLGA NPs, Lf-conjugated TAX-loaded NPs, pure PLGA, pure PEG, and pure Lf. The sample solutions/suspensions were directly deposited onto the ATR crystal, followed by drying under a nitrogen stream. Spectra were acquired in absorbance mode using 64 scans at a resolution of 4 cm^−1^, over a wavenumber range of 4000–400 cm^−1^, for both the sample and background observations. Data processing was carried out in GraphPad Prism (version 9.1.2, GraphPad Software, San Diego, CA, USA).

#### 2.5.4. Transmission Electron Microscopy

Transmission electron microscopy (TEM) was used for NPs’ morphological characterization. For sample preparation, 5 µL of NP suspension containing 0.010 mg of NPs was deposited onto a carbon-coated copper grid (Formvar/Carbon-400 mesh Copper, Agar Scientific, Rotherham, UK) and allowed to adsorb for 5 min. The excess liquid was gently blotted with filter paper, and the grid was subsequently stained with a 2% (*w*/*v*) uranyl acetate solution in ultrapure water for 45 s. After drying, the NP sample was then observed in TEM to evaluate its morphology and confirm its size. Images were acquired using a Jeol JEM 1400 electron microscope (Jeol, Ltd., Tokyo, Japan), operated at an accelerating voltage of 80 kV.

### 2.6. In Vitro Release Studies

A release study was conducted for 23 days to evaluate the release kinetics of the encapsulated drug from the developed Lf-conjugated and non-conjugated TAX-loaded NPs under simulated physiological conditions. To better simulate the slightly acidic microenvironment of solid tumors, the release medium consisted of PBS (0.01 M) with the pH adjusted to 6.3 using HCl (0.1 M). Briefly, NPs containing 0.76 mg of TAX were dispersed in 2.8 mL of PBS and incubated at 37 °C under gentle stirring to ensure continuous agitation throughout the experiment (100 rpm).

Aliquoted samples were collected at defined time intervals and centrifuged at 14,500 rpm for 30 min to separate the NPs from the released TAX. The amount of drug present in the supernatant was determined by UV–Vis spectroscopy (Synergy 2 Microplate Reader, BioTek, UK) at 276 nm, based on a calibration curve previously prepared in PBS. Drug release was expressed as a cumulative percentage and represented as a function of time.

DLS and LDV measurements were performed to confirm the colloidal stability of the NPs under the simulated physiological conditions used for these experiments.

### 2.7. In Vitro Internalization Studies

#### 2.7.1. NP Uptake Quantification by Fluorescence Measurements

Fluorescence measurements were performed to evaluate the cell uptake of Lf-modified and non-modified NPs in human GB cell lines (U87, U251, and T98G) and healthy human astrocytes (NHA). For NP tracking, PLGA NPs were produced incorporating the fluorescent probe coumarin-6 (C6). Cells were seeded in 96-well tissue culture plates (F-shaped bottom, plasma-treated, VWR International GmbH, Dresden, Germany) at a density of 8000 cells per well and allowed to adhere for 24 h under the already described culture conditions. After cell adhesion, Lf-modified and non-modified C6-labeled NPs were diluted in DMEM and added to the cells at a final polymer concentration of 300 µg/mL in a total volume of 100 µL per well. The cells were incubated with the nanoformulations for two different periods (0.5 and 2 h). After incubation, the cells were rinsed with PBS to eliminate non-internalized NPs, followed by lysis by incubation with a solution of 0.1% (*v*/*v*) Triton X-100 in 0.1 M NaOH for 15 min at room temperature. The fluorescence intensity corresponding to internalized C6-loaded NPs was measured using a microplate reader (Synergy 2 Microplate Reader, BioTek, UK) at excitation and emission wavelengths of 430 nm and 485 nm, respectively.

#### 2.7.2. Competitive Blocking Assay of Lf-Binding Sites

To investigate if the internalization of Lf-conjugated NPs was mediated by specific Lf-binding receptors, a competitive receptor-blocking assay was performed. U87, U251, T98G, and NHA cells were seeded in 96-well plates (F-shaped bottom, plasma-treated, VWR International GmbH, Dresden, Germany) at a density of 8000 cells per well and allowed to adhere for 24 h. Prior to NP exposure, cells were pre-incubated with 100 µL of increasing concentrations of free Lf diluted in DMEM (0 to 5 mg/mL) for 1 h, aiming to saturate the Lf-binding sites at the cell surface. Following the incubation period, the cell media containing unbound Lf was removed, and cells were incubated with new culture media with Lf-conjugated or non-conjugated C6-loaded PLGA NPs for 2 h, added at a final polymer concentration of 300 µg/mL in a total volume of 100 µL per well. After incubation, the cells were rinsed with PBS to remove non-internalized NPs and subsequently lysed for fluorescence quantification, as detailed in Section 2.7.1.

### 2.8. In Vitro Cytotoxicity Studies

#### 2.8.1. Antiproliferative Activity of TAX-Loaded PLGA NPs

The antiproliferative activity of TAX-loaded PLGA NPs, either Lf-conjugated or non-conjugated, was evaluated using the SRB colorimetric assay. The cytotoxic of NPs was compared with those of free TAX. Briefly, cells (U87, U251, T98G and NHA) were seeded in 96-well tissue culture plates (F-shaped bottom, plasma-treated, VWR International GmbH, Dresden, Germany) at a density of 1000 cells per well and allowed to adhere under the previously described culture conditions. After cell adhesion, cells were treated with increasing concentrations of free TAX, non-conjugated TAX-loaded PLGA NPs, and Lf-modified TAX-loaded PLGA NPs, diluted in cell media (DMEM) at final TAX concentrations ranging from 1 to 2000 µM (total volume per well was 100 µL). Additionally, to assess the biocompatibility of the nanovehicle matrix, the cells were also exposed to unloaded PLGA and LF-conjugated PLGA NPs at polymer concentrations ranging from 50 to 300 µg/mL. Untreated cells were used as negative controls in all experiments. Following a 72-h incubation period, cells were fixed with 10% (*w*/*v*) TCA at 4 °C for 1 h, then stained with 0.4% (*w*/*v*) SRB for 20 min. Unbound dye was removed by rinsing with 1% (*v*/*v*) acetic acid, and the plates were left to dry at room temperature. The protein-bound dye was subsequently dissolved in 10 mM Tris buffer, and absorbance was recorded at 560 nm using a microplate reader (Synergy 2, BioTek, UK). Cell viability was calculated as a percentage relative to untreated controls. Dose–response curves were plotted, and IC_50_ values for TAX were obtained by applying non-linear regression analysis in GraphPad Prism (version 9.1.2, GraphPad Software, San Diego, CA, USA).

#### 2.8.2. Ability of TAX-Loaded PLGA NPs to Sensitize GB Cells to TMZ

The potential of Lf-modified and non-modified TAX-loaded PLGA NPs to increase the sensitivity of GB cells to the alkylating agent TMZ was assessed using the SRB assay. Cells (U87, U251, T98G) were seeded in 96-well tissue culture plates (F-shaped bottom, plasma-treated, VWR International GmbH, Dresden, Germany) at a density of 1000 cells per well and allowed to adhere for 24 h. The cells were then treated with TMZ, diluted in DMEM to obtain final concentrations ranging from 0.1 to 5000 µM, either alone or in combination with non-conjugated or Lf-conjugated TAX-loaded PLGA NPs at a fixed TAX concentration (total volume per well was 100 µL). The concentration of NPs used in these experiments was selected based on antiproliferative assay results, corresponding to the IC_80_ value determined for each cell line. Untreated cells were included as negative controls. After a 72-h incubation period, the SRB assay was performed as described in Section 2.8.1.

### 2.9. Statistical Analysis

Statistical analysis was performed using the *t*-test to evaluate significant differences between sample groups, with a significance level set at *p* < 0.05 (95% confidence interval). The results are given as the mean and standard deviation (SD).

## 3. Results and Discussion

### 3.1. ANOVA Statistical Analysis of the Factorial Design

A full factorial design was implemented to optimize the NPs’ preparation protocol using Design Expert^®^ software. Among DOE approaches, factorial designs evaluate all possible combinations of selected variables simultaneously, allowing for the assessment of individual and interaction effects. This method is widely applied in NP research for robust optimization of formulation parameters and was the design selected and employed in the present work [27]. Presented in Table S1 (Supplementary Information File) is the summary of the DOE and the obtained responses. Additionally, Tables S2–S6 present the ANOVA results obtained for the five responses in the factorial design experiment, i.e., particle size, PDI, zeta potential, EE, and LC.

For all responses, the model terms were statistically significant (*p* < 0.05), confirming their adequacy to describe the system behavior. A linear model proved suitable for representing the behavior of all studied variables, with a good fit between the predicted and experimental values. Therefore, all responses were included in the model, and regression coefficients (RCs) were calculated for all. These coefficients were used to establish regression equations (Equations (S1)–(S5)) capable of predicting each individual response as a function of the experimental variables. The sign of each RC indicates the direction of the effect of each factor on the response, allowing for a detailed understanding of their influence. To better visualize the effects of variables on the responses, 2D contour plots were generated and are presented in the Supplementary Information File (Figures S1–S4).

The size of the produced NPs varied between 95.2 (run 1) and 214.9 nm (run 17) (Table S1). From the obtained regression equation, 2D contour plots, and ANOVA statistical results (Equation (S1), Figure S1, and Table S2), the number of sonication cycles, PVA concentration, and amount of PLGA were found to have a statistically significant impact on particle size (*p* < 0.05). As shown in Figure S1A, increasing both the number of sonication cycles and PVA concentration led to a reduction in NP size, indicating that these two variables have a negative effect on this response. This trend may be attributed to higher energy input from prolonged sonication, which enhances droplet disruption, and to increased surfactant availability, which stabilizes smaller particles during emulsification [28,29]. It was also observed that higher PLGA amounts resulted in larger NPs, showing that this variable has, in contrast, a positive effect on the NP size. This can be explained by the fact that an increase in PLGA content increases the viscosity of the organic phase, making it more resistant to shear forces during emulsification and decreasing efficiency in droplet breakup under sonication. As a result, larger initial droplets are formed and consequently larger NPs [30].

The obtained PDI values ranged from 0.051 (run 31) to 0.350 (run 18) (Table S1). By analyzing Table S3, it was observed that among all variables, only the EA/PVA ratio had a statistically significant effect on the PDI (*p* < 0.05). The obtained regression equation (Equation (S2)) shows that increasing the EA/PVA ratio led to a decrease in PDI values. This observation suggests that a greater proportion of organic solvent relative to the aqueous phase contributes to more homogeneous particle formation, possibly by reducing viscosity and enhancing droplet breakup, thereby limiting the formation of aggregates and promoting narrow size distributions [31].

The zeta potential values of the produced NPs varied from −7.6 (run 24) to 7.8 mV (run 22) (Table S1). Zeta potential was significantly affected by PVA concentration, PLGA amount, and EA/PVA ratio, all with p-values below 0.05 (Table S4). It was observed that zeta potential values became progressively more negative with increasing PLGA content and increasing PVA concentration, in agreement with previously reported in the literature [32] (Equation (S3) and Figure S2). These effects may be explained by the higher PLGA content, which can increase the exposure of carboxylic acid terminal groups at the NP surface, thereby enhancing the overall negative surface charge. Additionally, the elevated PVA concentration may result in greater adsorption of hydroxyl-rich surfactant molecules at the interface, further contributing to the negative charge of the particles [33]. Additionally, it was verified that higher EA/PVA ratios generated less negative zeta potential values. This effect can be attributed to the lower availability of PVA in the aqueous phase, resulting in reduced adsorption of hydroxyl-containing surfactant molecules at the NPs surface [33].

The EE values varied from 1.44% (run 20) to 84.42% (run 17) (Table S1). From the obtained regression equation, 2D contour plots, and ANOVA statistical results (Equation (S4), Figure S3, and Table S5), it was concluded that the EE was significantly influenced by all variables (*p* < 0.05). It was verified that higher PLGA concentrations and higher EA/PVA ratios led to higher EE values, showing a positive effect of both variables. This can be attributed to the greater availability of polymer to encapsulate the drug and to the increased proportion of organic solvent, which may improve emulsification and reduce drug loss to the aqueous phase during formulation [34]. Additionally, the EE was higher at lower PVA concentrations and lower sonication cycles. These effects can be explained by reduced surfactant concentration, which may limit drug solubilization into the external phase, while excessive sonication could destabilize the emulsion or promote drug leakage, ultimately reducing EE [35].

The LC of the produced NPs ranged from 0.06% (run 5) to 8.66% (run 10). (Table S1). It was observed that only the amount of PLGA and the EA/PVA ratio had a statistically significant effect on the LC (*p* < 0.05) (Table S6). As shown in Equation (S5) and Figure S4, increasing the PLGA amount was associated with a reduction in LC. This negative effect likely reflects the dilution of drug content relative to the total mass of polymer, which increases disproportionately when the drug dose remains fixed [36]. Moreover, lower EA/PVA ratios, while beneficial for EE, were shown to reduce the LC percentage.

### 3.2. Optimization of TAX-Loaded PLGA NPs Preparation

Based on the statistical analysis of the experimental results, the optimal formulation parameters were then determined to define the most suitable conditions for NP preparation. The optimization protocol was carried out using Design-Expert^®^ software, where the independent variables were restricted to the experimental ranges established during the factorial design, and specific goals were defined for each response variable. For the NPs’ size, the goal was to maintain the values within a defined range between 120 and 200 nm, ensuring compatibility with systemic circulation and tumor accumulation. The PDI was set with the goal of minimizing its value, with an upper limit of 0.2. For the zeta potential, the goal was also set to minimize it without defining a specific range. The goal for EE was set to maximize it, ensuring the highest possible drug encapsulation, while guaranteeing a lower threshold above 40%. Regarding LC, the goal was also set to maximize it without defining a specific range. The optimal formulation was selected based on all response variables considered together, and not by prioritizing a single response. Based on these predefined goals, the software generated the optimal formulation conditions, corresponding to the following experimental values for the independent variables: one sonication cycle, 1.0% (*w*/*v*) PVA, 50.0 mg of PLGA, and an EA:PVA volume ratio of 0.75.

To assess the validity of the model predictions and the predicted formulation parameters, 6 independent batches of the optimized formulation were prepared. The results obtained, along with the corresponding predicted values for comparison, including confidence intervals (CI), are presented in Table 2 as mean ± standard deviation (*n* = 6).

Analysis of Table 2 shows that the mean experimental responses for the optimized TAX-loaded NPs fall within the predicted intervals generated by the software, reflecting an appropriate mathematical fit and predictive accuracy of the model.

Furthermore, the resulting NPs exhibited desirable physicochemical properties for systemic administration, particularly for brain delivery purposes. Their size, ranging between 100 and 200 nm, is ideal for passive tumor targeting via the Enhanced Permeability and Retention (EPR) effect, while also avoiding rapid clearance mechanisms and limiting off-target accumulation [37]. Furthermore, the low PDI indicates a monodisperse and stable colloidal system. Although the zeta potential of the optimized formulation was close to neutrality, this does not necessarily indicate poor colloidal stability. PVA and PEG can provide steric stabilization, helping prevent particle aggregation. In fact, stability studies performed under storage conditions (aqueous suspension, 4 °C) demonstrated that the NPs remained stable for up to 36 days (Figure S6A in the Supplementary Information File). The near-neutral zeta potential observed may also be attributed to the presence of TAX within the NPs’ structure, which can partially mask the surface charge of the polymer matrix. Altogether, these features support the suitability of the developed NPs for application in GB therapy.

These results are in line with previously reported PLGA-based nanosystems, where similar size ranges and low PDI values are commonly associated with improved circulation profiles and tumor accumulation in vivo [38,39]. The ability to simultaneously achieve adequate size, low dispersity, and high encapsulation further highlights the robustness of the optimization approach. Overall, the obtained formulation parameters and resulting NP characteristics are consistent with those described in the literature for nanocarriers intended for brain-targeted drug delivery.

### 3.3. Lf Conjugation

To enhance the targeting capability of the NPs, Lf was conjugated onto the surface of the optimized TAX-loaded formulations. Table 3 summarizes the physicochemical properties before and after the conjugation process, including size, PDI, and zeta potential, as well as EE and CE of Lf.

The high CE value of 65.0% indicates a successful surface functionalization process, corresponding to an average of approximately 130 Lf molecules per NP. Additionally, the amount of drug lost during the conjugation process was quantified to assess potential leakage of TAX from the NPs. It was observed that following surface modification with Lf, no significant drug release was detected during the conjugation process, which is highly favorable. In fact, the EE remained consistently above 65%, with no statistically significant difference (*p* > 0.05), confirming that the conjugation process did not compromise the NPs’ drug encapsulation.

Statistical analysis also demonstrated that upon conjugation with Lf, the NPs’ mean size did not increase (*p* > 0.05). Some authors reported size increases following ligand attachment to PLGA-based nanocarriers [40]. However, in this work, this was not observed. The mean hydrodynamic diameter after conjugation remained within the optimal range for EPR in tumor tissues. The PDI values remained low and comparable before and after conjugation, indicating that the NPs maintained their homogeneity following surface modification with no statistically significant difference observed (*p* > 0.05).

On the contrary, a trend toward a more negative zeta potential following Lf conjugation was observed, with a mean difference of approximately 15.5 mV. These findings suggest that the conjugation process altered the interfacial properties of the NPs, indicating that the conjugation was successful. Similar behavior was previously reported by Huang et al. (2013) [41], who documented a shift from –0.60 mV to –25.4 mV upon conjugation of Lf to PEGylated liposomes. The authors attributed this increase in negative charge to changes in the surface architecture and electrostatic environment induced by the conjugation process. Specifically, they noted that the presence of Lf may expose negatively charged domains or reorganize the hydration and ionic layers surrounding the particles. This highly notable change reinforces the conclusion that Lf was successfully conjugated to the NPs surface.

**Figure 1:**
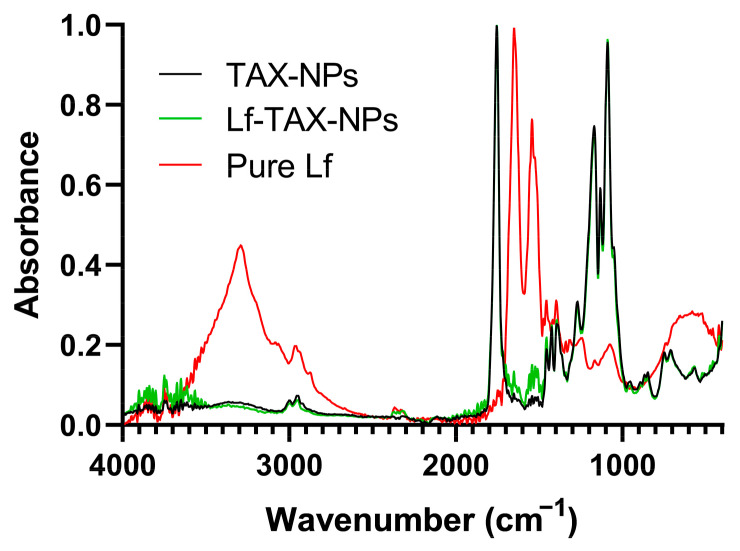
ATR-FTIR absorbance spectra of optimized Lf-conjugated and non-conjugated TAX-loaded PLGA NPs and pure Lf.

Additionally, ATR-FTIR spectroscopy was employed to confirm the successful surface modification of the developed PLGA NPs. ATR-FTIR spectra were recorded for Lf-conjugated and non-conjugated TAX-loaded NPs, and pure Lf as a control (Figure 1).

As verified in Figure 1, Lf exhibits characteristic amide I (1662–1637 cm^−1^) and amide II (1530–1525 cm^−1^) bands, along with a C–O–C vibration near 1093 cm^−1^. In the present study, the ATR-FTIR spectrum of Lf-conjugated NPs showed a subtle but consistent increase in absorption at ~1650 cm^−1^ and ~1530 cm^−1^ compared to non-conjugated TAX-loaded PLGA, suggesting surface conjugation of the protein. Additionally, a very slight increase in absorbance was observed in the broad O–H/N–H stretching region (3200–3400 cm^−1^), which may be indicative of the presence of Lf conjugated to the NP’s surface. The weak spectral contribution of Lf may also be attributed to its relatively low concentration compared to PLGA in the formulation, which may limit its detectability in ATR-FTIR [42]. ATR-FTIR analysis was also performed to confirm PEG–PLGA conjugation. The corresponding spectra, including comparison with pure PEG and pure PLGA are provided in the Supplementary Information (Figure S5). Due to the low PEG:PLGA ratio and overlapping spectral features, only subtle differences were observed.

Moreover, NPs’ conjugation with Lf did not impact the colloidal stability of the NPs. In fact, stability studies performed under storage conditions (aqueous suspension, 4 °C) demonstrated that the Lf-conjugated NPs remained stable for up to 36 days (Figure S6B in the Supplementary Information File).

These results are in line with what has been reported for Lf-functionalized systems, where conjugation does not significantly affect NPs’ stability, despite changing the surface charge, reflecting changes in the NP interface [43]. Maintaining colloidal stability is important to preserve circulation and tumor accumulation. At the same time, Lf conjugation is expected to enhance cellular uptake through receptor-mediated mechanisms, which is particularly relevant in GB. Overall, these results support the potential of the developed NPs for targeted delivery.

### 3.4. Morphological Analysis

**Figure 2:**
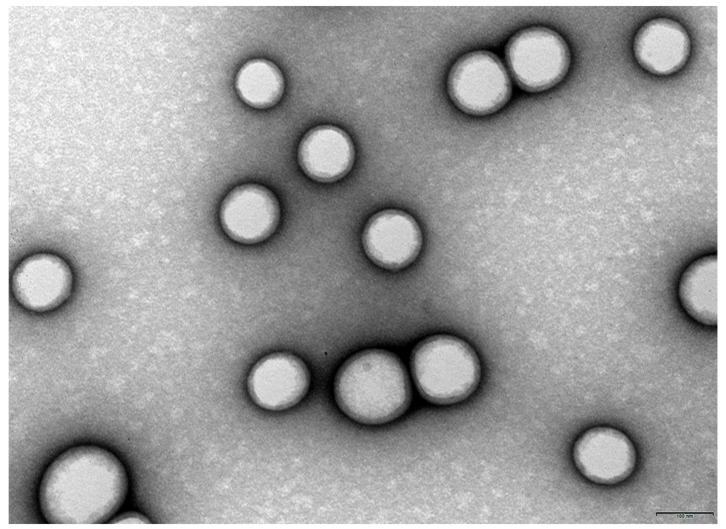
TEM image for morphological characterization of TAX-loaded PLGA NPs, showing a high density and homogeneous dispersion of spherical NPs. The image reveals well-defined, smooth-surfaced spherical NPs with uniform size distribution (scale bar = 100 nm).

TEM was employed to confirm the morphological characteristics and size distribution of the NPs. The images obtained are presented in Figure 2.

Figure 2 shows a monodisperse population of spherical NPs with uniform size distribution. The particles appear well-separated, with no evidence of aggregation and with a morphological homogeneity desirable for biomedical applications. Additionally, the image highlights their smooth surface and spherical morphology. Thus, the NPs were demonstrated to be well-defined and structurally intact. Overall, the TEM images confirmed that the developed NPs exhibit appropriate morphological features, including spherical shape, smooth surface, and uniform distribution.

### 3.5. TAX Release Behavior and Colloidal Stability Under Physiological Conditions

**Figure 3:**
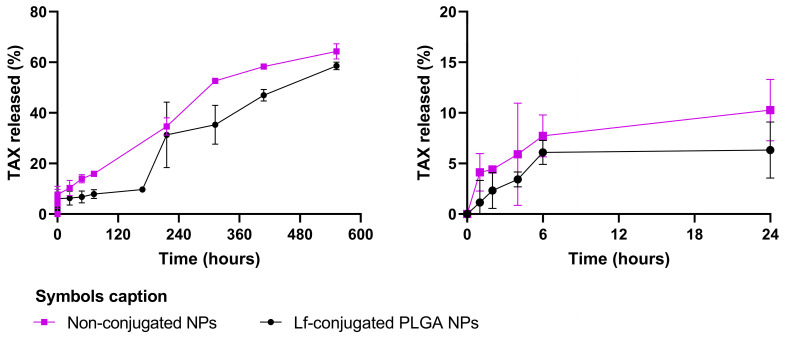
In vitro release profile of TAX from PLGA NPs over 23 days (552 h) in PBS (pH = 6.3, 0.01 M) at 37 °C. On the right side of the figure, a magnified view of the first 24 h of the experiment is presented. The results are presented as mean ± SD (*n* = 3). Error bars represent SD.

The release behavior of TAX from Lf-modified NPs was investigated in vitro under simulated physiological conditions (PBS 0.01 M, pH 6.3) at 37 °C over 23 days, mimicking the acidic tumor microenvironment. To assess the effect of Lf conjugation on the release behavior, TAX release from non-conjugated NPs was also studied as a control. Figure 3 illustrates the TAX release profile of the developed NPs.

As shown in Figure 3, an initial burst release was observed for both formulations, with approximately 8 ± 2% and 16 ± 0.4% of TAX released from Lf-conjugated and non-conjugated NPs after 72 h, respectively. This behavior is characteristic of PLGA NPs and is typically attributed to the desorption of drug molecules adsorbed onto or weakly associated with the NPs’ surface upon contact with the aqueous medium. However, the magnitude of the burst release was lower than that reported by other authors, suggesting a low amount of TAX adsorbed onto the NP surface.

Interestingly, as observed during NP characterization, the non-conjugated NPs exhibited a nearly neutral zeta potential, suggesting a higher degree of drug adsorption at the surface. However, part of the drug may be located near the NP surface yet still embedded within the polymer matrix. This intermediate positioning could influence the surface charge without necessarily leading to immediate or rapid release.

Subsequently, the release rate remained relatively slow and sustained for both formulations, reaching cumulative values of approximately 59 ± 2% and 64 ± 3% after 23 (552 h) days for Lf-conjugated and non-conjugated NPs, respectively. Hydrophobic drugs such as TAX tend to exhibit prolonged release profiles from PLGA NPs due to their low aqueous solubility and limited contribution to polymer swelling, degradation, and diffusion processes [44,45]. The release study was limited to 23 days because the release rate slowed after this period, and extending it further would not be representative of in vivo conditions.

Furthermore, TAX release was significantly faster for the non-conjugated NPs (*p* < 0.05), suggesting that Lf conjugation influences the drug release behavior. Similar observations have been reported by other authors, who demonstrated that the surface conjugation of proteins such as Lf or transferrin (Tf) onto PLGA NPs can affect drug release. This effect may be attributed to the formation of an additional surface layer that acts as a diffusion barrier, reducing water penetration and slowing drug diffusion from the polymeric matrix [46,47].

The selection of pH 6.3 was intended to represent mildly acidic conditions relevant to the GB tumor microenvironment. Under these conditions, the developed NPs exhibited a sustained release profile, supporting their suitability for application in this context. While other studies often evaluate release at physiological pH (7.4), several works have also investigated PLGA-based systems under mildly acidic conditions (pH ~6–6.5) to better mimic the tumor microenvironments [48].

**Figure 4:**
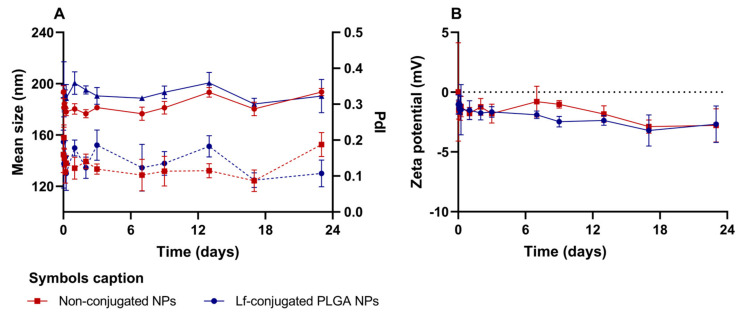
Colloidal stability of Lf-conjugated and non-conjugated TAX-loaded PLGA NPs in simulated physiological conditions (PBS, pH 6.3, 37 °C), evaluated for 23 days, in terms of changes in (**A**) mean size (full lines) and PDI values (dotted lines); (**B**) zeta potential. All data are presented as mean ± SD (*n* = 3). Error bars represent SD.

Stability under simulated physiological conditions was investigated for the developed NPs by monitoring variations in zeta potential, size and PDI over 23 days. The results obtained are summarized in Figure 4.

The results showed that the mean size of the NPs did not significantly change from the beginning to the end of the experiment (day 23) (*p* > 0.05), indicating that both nanoformulations did not undergo significant aggregation or swelling under the tested conditions. Despite that, fluctuations in PDI values were observed over time for both Lf- and non-conjugated NPs. As these changes were not accompanied by a significant increase in size, these may be associated with subtle shifts in population homogeneity and interparticle interactions rather than aggregation.

Additionally, the zeta potential values of both Lf-conjugated and non-conjugated NPs did not suffer significant changes over time (*p* > 0.05), indicating electrostatic stability under the tested conditions. However, it was observed that the Lf-conjugated NPs exhibited zeta potential values closer to neutrality under release conditions, which was not observed after production (Table 3). This difference is due to a change in the dispersion medium, and it can be attributed to the buffering effect of PBS, which neutralizes the surface charges on the NPs. The interaction between the free charges in PBS and the negative charges on the NP surface leads to a zeta potential less negative and closer to zero when the particles are in suspension in PBS.

Overall, the obtained release and stability profiles are consistent with those reported for PLGA-based systems, supporting the suitability of the developed NPs for controlled drug delivery applications while maintaining particle integrity.

### 3.6. Receptor-Mediated Cellular Internalization of Lf-PLGA NPs

**Figure 5:**
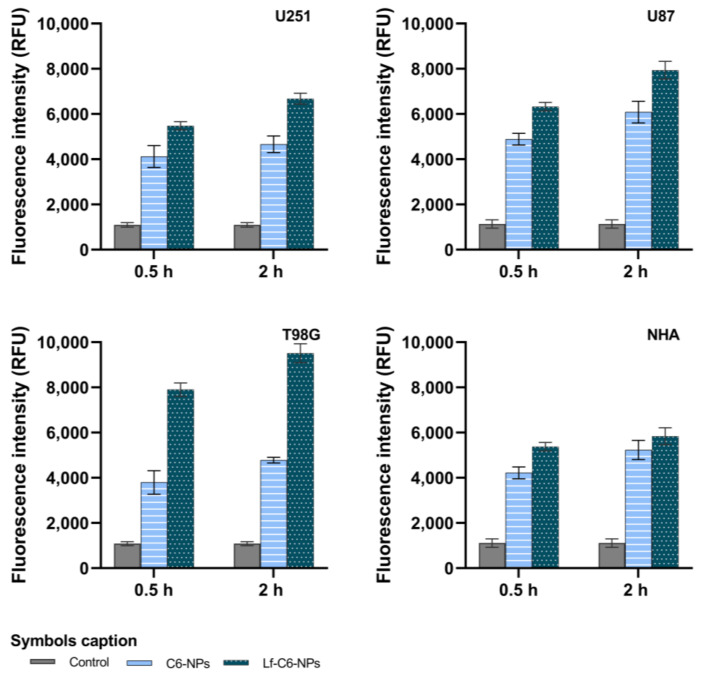
Fluorescence-based quantification of C6-labeled NP uptake after 0.5- and 2-h treatments in human GB cells (U87, U251, and T98G) and healthy human astrocytes (NHA). Control bars represent untreated cell autofluorescence. The results are presented as mean ± SD (*n* = 3). Error bars represent SD.

The effect of Lf-conjugation on the cellular uptake of the developed PLGA NPs was evaluated in three human GB cell lines (U87, U251, and T98G) and in NHA using C6 as a fluorescent tracer. The influence of incubation time was also assessed after 0.5 and 2 h of exposure, and the obtained results are presented in Figure 5.

It was observed that NPs’ internalization is time-dependent in all tested cell lines, with significantly higher uptake levels observed after 2 h compared to 0.5 h, regardless of the NPs’ conjugation (*p* < 0.05). These results were expected since a prolonged exposure favors increased NP–cell interactions required for internalization by an endocytic uptake mechanism.

Furthermore, surface conjugation with Lf significantly enhanced NP internalization in all studied cell lines and incubation times when compared to non-conjugated PLGA NPs (*p* < 0.05). However, this effect was more pronounced in GB cells than in healthy astrocytes, demonstrating the potential of NPs’ conjugation with Lf to improve tumor selectivity. Among the three studied tumor cell lines, T98G cells exhibited the most pronounced increase in NP uptake due to ligand conjugation. In fact, after 2 h of incubation, Lf-conjugated NPs showed an increase in uptake of approximately 43% in U251 cells, 30% in U87 cells, and 99% in T98G cells relative to non-conjugated NPs. In contrast, only a slight increase of about 12% was observed in healthy astrocytes. This enhanced uptake observed in tumor cells can be explained by the overexpression of Lf-binding receptors, such as LRP1 (CD91), in GB cells compared to healthy astrocytes [49].

**Figure 6:**
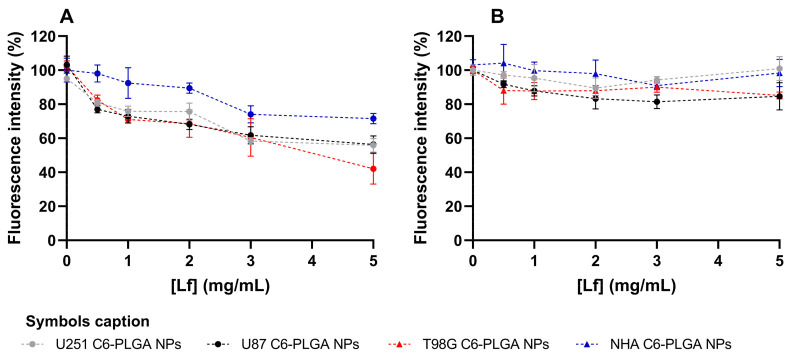
Receptor blocking experiment and its effect on the internalization of C6-labeled PLGA NPs in human cells (U87, U251 and T98G, and NHA). Following pre-treatment with increasing concentrations of free Lf for 1 h to saturate cell surface Lf-binding sites, cells were incubated for 2 h with Lf-modified or non-conjugated C6-labeled NPs. (**A**) Data for Lf-modified NPs. (**B**) Data for non-modified NPs. All data are presented as mean ± SD (*n* = 3). Error bars represent SD.

To further evaluate the mechanism regulating NP uptake, a receptor blocking assay was performed. The cells were pre-treated with increasing concentrations of free Lf before incubation with Lf-conjugated or non-conjugated PLGA NPs. The results obtained are shown in Figure 6.

The results demonstrated a clear dose-dependent reduction in the uptake of Lf-conjugated NPs as the concentration of free Lf increased. In contrast, the internalization of non-conjugated NPs was not significantly affected by Lf pre-treatment even at the highest tested Lf concentration (5 mg/mL) (*p* > 0.05). These findings suggest that NP uptake occurs in fact by an endocytosis mechanism involving Lf-binding receptors.

Additionally, this inhibitory effect was more pronounced in GB cells than in healthy astrocytes, further demonstrating the role of Lf-binding receptor-mediated endocytosis in tumor cells. For example, in T98G cells, pre-treatment with Lf at the highest tested concentration (5 mg/mL) reduced the uptake of Lf-conjugated PLGA NPs to approximately 42%, while in NHA cells, conjugated NPs exhibited only a slight reduction in uptake, remaining at approximately 72% for the same tested concentration.

In conclusion, these findings support the use of Lf as an effective targeting ligand to improve tumor selectivity. Considering the growing evidence that passive accumulation mechanisms such as the EPR effect may play a limited role in tumor cell targeting, NP conjugation with Lf is a promising active targeting strategy to promote selective NP uptake in GB cells. These results are in line with what has been reported for Lf-functionalized nanosystems, where enhanced uptake is attributed to receptor-mediated endocytosis involving Lf-binding receptors such as LRP1 [14]. The more pronounced effect observed in GB cells further demonstrates the relevance of this targeting strategy, as these receptors are known to be overexpressed in tumor cells [49]. Overall, these findings reinforce the potential of Lf conjugation to promote selective NP internalization and improve targeting efficiency in GB.

### 3.7. Antiproliferative Efficacy of TAX-Loaded PLGA NPs

**Figure 7:**
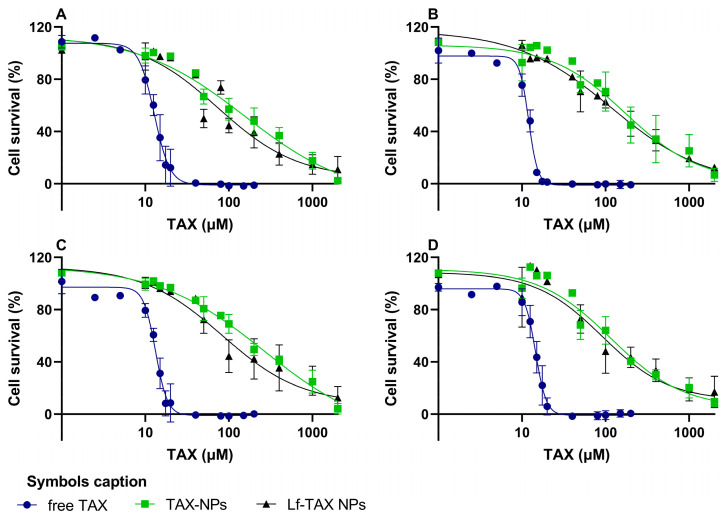
Cell survival quantification by SRB assay after 72 h treatment with free TAX, TAX-loaded Lf-conjugated and non-conjugated PLGA NPs on the cell survival of (**A**) U251, (**B**) U87, (**C**) T98G and (**D**) NHA cells. Cell survival is presented as a percent (% = Absorbance of treated cells/Absorbance of control cells × 100). Data is given as mean ± SD (*n* = 3). Error bars represent SD.

The antiproliferative efficacy of the developed Lf-conjugated TAX-loaded NPs was evaluated in three human GB cell lines (U87, U251, and T98G) and in NHAs and compared with free TAX. To assess the effect of Lf-conjugation, non-conjugated NPs were also evaluated. The results obtained after 72 h of treatment are presented in Figure 7.

The survival inhibition curves demonstrated a dose-dependent decrease in cell viability for all treatments and cell lines. Additionally, to confirm that the observed therapeutic effect was associated with TAX and to evaluate the biocompatibility of the materials used, unloaded PLGA NPs, both Lf-conjugated and non-conjugated, were also tested in all four cell lines. No significant decrease in cell viability was observed within the tested concentration range (Figure S7 in the Supplementary Information File), confirming the biocompatibility of the developed NPs.

Furthermore, IC_50_ values were determined by fitting a non-linear regression model to the dose–response curves in Figure 7, and the obtained values are summarized in Table 4.

Free TAX exhibited a similar antiproliferative effect in all tested cell lines, with IC_50_ values ranging between 12 and 14 µM. These values are in agreement with those reported in the literature for GB cells, where IC_50_ values typically range from 5 to 50 µM, depending on the experimental conditions and cell line [6,50]. Also, comparable toxicity was observed in healthy astrocytes, indicating that free TAX does not exhibit selectivity toward tumor cells under the tested conditions.

Furthermore, based on the obtained IC_50_ values, free TAX appears to be more efficient than both TAX-loaded NP formulations, independently of surface functionalization (*p* < 0.05). However, as demonstrated in the release studies, only approximately 8% and 16% of the encapsulated TAX were released within 72 h from Lf-conjugated and non-conjugated NPs, respectively, which corresponds to the duration of the cytotoxicity assay. Therefore, the total encapsulated drug is not fully available to exert its biological effect during the duration of the experiment.

To allow a more accurate comparison with free TAX, estimated IC_50_ values were calculated considering the fraction of drug released at 72 h. These estimated values are also presented in Table 4. The estimated IC_50_ values of Lf-conjugated NPs were comparable to those of free TAX in all tested cell lines, and in the case of U251 cells, were even significantly lower (*p* < 0.05). For the non-conjugated NPs, the estimated IC_50_ values were within the same order of magnitude as free TAX, although slightly higher. These results show the importance of considering drug release kinetics when evaluating the efficacy of nanoformulations. Although free TAX appears more efficient, this is mainly related to the limited fraction of the drug released during the experimental timeframe. This behavior has also been reported by other authors, who showed that the apparent lower efficacy of NP-based systems is often associated with sustained release profiles rather than reduced intrinsic activity [51,52].

Additionally, these findings suggest that Lf conjugation does enhance the therapeutic efficacy of the nanoformulation. In fact, both the real and release-estimated IC_50_ values were lower for Lf-conjugated NPs compared to non-conjugated NPs in all tested cell lines. These results demonstrate that surface functionalization with Lf potentiates the antiproliferative effect of the developed TAX-loaded NPs. Additionally, this enhancement was more pronounced in GB cell lines than in healthy astrocytes. For example, in NHA cells, the IC_50_ value of non-functionalized NPs was only 17% higher than that of Lf-conjugated NPs, whereas in T98G cells, the difference reached 59% (*p* < 0.05). These results are in agreement with the observation of the uptake studies, which showed that Lf conjugation significantly enhanced NPs’ internalization in GB cells, particularly in T98G cells, while a less pronounced effect was observed in healthy astrocytes. When considered together with the uptake results, these findings suggest that the improved internalization observed for Lf-conjugated NPs contributes directly to their enhanced antiproliferative effect. This effect is particularly evident in GB cells, where Lf-mediated uptake is more pronounced, supporting the role of Lf as an effective targeting ligand to improve intracellular drug delivery.

In sum, these experiments demonstrate that Lf functionalization improves intracellular uptake of TAX-loaded NPs preferentially in tumor cells, supporting the potential of this strategy to enhance therapeutic selectivity.

### 3.8. In Vitro Sensitization of GB Cells to TMZ by TAX-Loaded PLGA NPs

**Figure 8:**
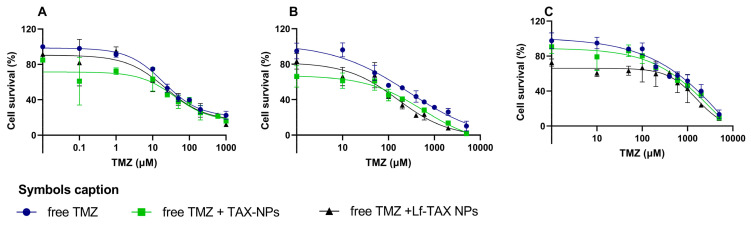
Cell survival quantification by SRB assay after 72 h treatment with free TMZ and combination therapy of free TMZ with TAX-loaded Lf-conjugated or non-conjugated PLGA NPs in (**A**) U251, (**B**) U87, and (**C**) T98. The NPs were used at a fixed concentration corresponding to the IC_80_ value previously determined for each cell line. Cell survival is presented as a percentage (% = Absorbance of treated cells/Absorbance of control cells × 100). Data are given as mean ± SD (*n* = 3). Error bars represent SD.

To evaluate whether the developed nanoformulations could enhance the sensitivity of GB cells to alkylating agents, a combination therapy assay was performed. TMZ was selected because it is the gold standard treatment for GB. For these experiments, three human GB cell lines (U87, U251, and T98G) were used, as they present different levels of expression of the DNA repair protein MGMT, which is known to influence cellular sensitivity to alkylating agents. The results obtained are presented in Figure 8.

Furthermore, IC_50_ values were determined by fitting a non-linear regression model to the dose–response curves in Figure 8, and the obtained values are summarized in Table 5.

The IC_50_ values for free TMZ are consistent with those reported in the literature for these GB cell lines, which exhibit different sensitivities to alkylating agents due to their distinct MGMT expression levels. Among the studied cell lines, T98G has been reported to exhibit the highest MGMT expression, while U251 presents the lowest levels [53].

In the U87 cell line, which combined TMZ with TAX-loaded NPs, a significant reduction in IC_50_ was observed. In particular, IC_50_ values decreased from 250 ± 21 to 92 ±10 and 86 ± 13 µM when TMZ was combined with non-conjugated and Lf-conjugated NPs (*p* < 0.05), respectively. These results indicate that the presence of the nanoformulations enhances the sensitivity of U87 cells to TMZ.

A similar effect was observed for the highly TMZ-resistant T98G cell line. In this case, treatment with TMZ alone resulted in an IC_50_ of 1162 ± 48 µM. When combined with TAX-loaded NPs, the IC_50_ values decreased to 810 ± 33 and 645 ± 26 µM for the non-conjugated and Lf-conjugated TAX-loaded NPs, respectively (*p* < 0.05).

In contrast, for the TMZ-sensitive U251 cell line, the combination treatment resulted in the smallest change in IC_50_ compared with TMZ alone. This can be explained by the low MGMT expression in this cell line, which already exhibits relatively high sensitivity to alkylating agents.

Thus, these findings suggest that TAX-loaded PLGA NPs can act as chemosensitizers, enhancing the responsiveness of GB cells to TMZ treatment. In fact, the effect was more pronounced in the TMZ-resistant cell lines, particularly when Lf-functionalized NPs were used. Thus, this work demonstrates the potential of TAX-loaded NPs to modulate the response of GB cells to standard chemotherapy, particularly in resistant cells. The more pronounced effect observed in T98G cells suggests that this strategy may be especially relevant in tumors with high MGMT expression, where conventional TMZ therapy is less effective. By combining sustained drug delivery with enhanced cellular uptake, the developed nanoformulations may overcome intrinsic resistance mechanisms. This suggests that combining drug repositioning strategies with nanotechnology-based approaches may be a promising strategy to improve therapeutic outcomes in GB cases associated with MGMT-mediated resistance.

## 4. Conclusions

Current GB treatments remain non-curative and are associated with poor prognosis and high mortality, largely because conventional regimens do not consistently overcome intrinsic chemoresistance mechanisms (including MGMT-associated resistance) and because the BBB severely restricts effective drug delivery to intracranial tumor tissue. Nanotechnology offers a promising strategy to address these barriers, enabling improved handling of poorly soluble drugs, controlled and sustained release, enhanced precision through ligand-mediated targeting, and reduced systemic exposure compared with free drug administration. In this work, an optimized PLGA NP formulation was developed to encapsulate TAX within a drug-repositioning framework and was further surface-engineered with PEG and LF to improve GB targeting. Comprehensive in vitro characterization supported the suitability of the engineered NPs for GB application by confirming appropriate physicochemical attributes, such as mean diameters below 200 nm, low PDI value, highly negative zeta potential values, and EE of about 69%. Furthermore, the developed NPs showed colloidal stability under storage and physiological conditions, and a sustained release profile compatible with prolonged drug availability. Cell uptake studies performed in GB cell lines and healthy astrocytes showed that Lf-functionalization enhanced NP internalization, particularly in tumor cells. Cytotoxicity assays further demonstrated that Lf-conjugated NPs improved the antiproliferative effect of TAX compared to non-functionalized formulations. Additionally, combination experiments indicated that the developed NPs were able to increase the sensitivity of GB cells to TMZ treatment. Overall, these findings indicate that PEG-Lf-decorated TAX-loaded PLGA NPs constitute a promising platform for GB therapy by integrating GB-oriented targeting design with controlled delivery, thereby supporting their further evaluation as a novel approach to improve therapeutic performance against this devastating disease.

## Figures and Tables

**Table 1:** Independent variables and corresponding levels used in the factorial design.

Factor	Variable	Unit	−1	0	+1
A	Number of sonication cycles	n.a.	1	4	7
B	PVA concentration	% (*w*/*v*)	1	3	5
C	Amount of PLGA	mg	10	30	50
D	EA: PVA ratio	n.a.	0.25	0.50	0.75

n.a.: not applicable.

**Table 2:** Software-predicted vs. experimental values for size, PDI, zeta potential, EE, and LC for the produced batches, with experimental data presented as mean ± SD (*n* = 6).

Parameter	Predicted Values (95% CI)	Experimental Values
Size (nm)	199.9 (181.7–218.1)	190 ± 17
PDI	0.13 (0.07–0.18)	0.18 ± 0.06
Zeta Potential (mV)	−0.24 (−3.57–3.08)	−2.7 ± 1.6
EE (%)	82.1 (66.5–97.8)	72.8 ± 3.8
LC (%)	1.60 (0.45–2.75)	1.5 ± 0.07

**Table 3:** Physicochemical characterization of NPs (optimal formulation) before and after Lf conjugation, namely results for DLS measurements of size (nm), PDI, and zeta potential (mV), as well as the obtained values for EE (%) and CE (%) after conjugation (*n* = 3).

	Size (nm)	PDI	Zeta Potential (mV)	EE (%)	CE (%)
Before Lf conjugation	190 ± 17	0.18 ± 0.06	−2.7 ± 1.6	72.8 ± 3.8	-
After Lf conjugation	193 ± 6	0.11 ± 0.04	−18.2 ± 6.8	68.6 ± 1.8	65.0 ± 7.0

**Table 4:** IC_50_ values determined by non-linear regression of the dose–response curves after 72 h of treatment with free TAX, TAX-loaded Lf-conjugated and non-conjugated PLGA NPs for the different studied human GB cells (U251, U87, T98G) and NHAs. In the table, the IC50 values, estimated based on the TAX released at 72 h, are also presented. Data is given as mean ± SD (*n* = 3).

IC_50_ (µM)					
	Free TAX	TAX-PLGA NPs		Lf-TAX-PLGA NPs	
		Real	Estimated	Real	Estimated
U251	14.0 ± 1.8	169 ± 62	27 ± 10	102 ± 30	8 ± 2
U87	11.8 ± 0.6	223 ± 94	36 ± 15	161 ± 29	13 ± 2
T98G	13.9 ± 1.6	252 ± 73	40 ± 12	158 ± 13	13 ± 1
NHA	14.3 ± 1.1	151 ± 38	24 ± 6	125 ± 45	10 ± 4

**Table 5:** IC_50_ values determined by non-linear regression of the dose–response curves after 72 h treatment with free TMZ and combination therapy of free TMZ with TAX-loaded Lf-conjugated or non-conjugated PLGA NPs for the different studied human GB cells (U251, U87, T98G). Data is given as mean ± SD (*n* = 3).

IC_50_ (µM)			
	Free TMZ	Free TMZ + TAX-PLGA NPs	Free TMZ + Lf-TAX-PLGA NPs
U251	35.3 ± 4.6	24.8 ± 1.8	25.0 ± 1.6
U87	250 ± 21	92 ±10	86 ± 13
T98G	1162 ± 48	810 ± 33	645 ± 26

## Data Availability

The original contributions presented in this study are included in the article/Supplementary Material. Further inquiries can be directed to the corresponding authors.

## Supplementary Materials

The following supporting information can be downloaded at https://www.mdpi.com/article/10.3390/polym18091055/s1: Table S1: Obtained responses for the 35 runs of the DOE, associated with each independent variable combination; Table S2: Results of the ANOVA statistical analysis of the DOE for response 1, Size. Table S3: Results of the ANOVA statistical analysis of the DOE for response 2, PDI. Table S4: Results of the ANOVA statistical analysis of the DOE for response 3, Zeta Potential. Table S5: Results of the ANOVA statistical analysis of the DOE for response 4, EE. Table S6: Results of the ANOVA statis-tical analysis of the DOE for response 5, LC. Regression Equations (S1)–(S5): Predictive models for size, PDI, zeta potential, EE, and LC; Figure S1. 2D contour plots illustrating the influence of selected formulation and process variables on the particle size of the PLGA NPs. The color gradient ranges from blue to red, indicating smaller to larger sizes, respectively. S1.A- Effect of the number of son-ication cycles (A) and PVA concentration (B). S1.B- Effect of the number of sonication cycles (A) and amount of PLGA (C). Figure S2. 2D contour plots illustrating the influence of selected formulation and process variables on the zeta potential of the PLGA NPs. The color gradient ranges from blue to red, indicating lower (more negative) to higher (less negative or positive) zeta potential values, re-spectively. S2.A - Effect of the PVA concentration (B) and amount of PLGA (C). S2.B - Effect of the PVA concentration (B) and EA/PVA ratio (D). Figure S3. 2D contour plots illustrating the influence of selected formulation and process variables on the EE of the PLGA NPs. The color gradient ranges from blue to red, indicating lower to higher EE values, respectively. S3.A - Effect of the amount of PLGA (C) and the EA/PVA ratio (D). S3.B - Effect of the number of sonication cycles (A) and the PVA concentration (B). Figure S4: 2D contour plot illustrating the influence of formulation and process variables on the LC of the PLGA NPs. The color gradient ranges from blue to red, repre-senting lower to higher LC values, respectively. The plot displays the combined effect of the amount of PLGA (C) and the EA/PVA volume ratio (D). Figure S5: ATR-FTIR absorbance spectra of pegylated TAX-loaded PLGA NPs, pure PLGA, pure PEG and pure TAX. Figure S6: Colloidal sta-bility of Lf-conjugated and non-conjugated TAX-loaded PLGA NPs in storage conditions (4 °C, aqueous suspension) evaluated for 36 days, in terms of changes in (A) mean size (full lines) and PDI values (dotted lines); (B) zeta potential. All data are presented as mean ± SD (n = 3). Error bars represent SD. Figure S7: Cell survival quantification by SRB assay after 72 h treatment with control unloaded Lf- conjugated and non-conjugated PLGA NPs on the cell survival of (A) U251, (B) U87, (C) T98G and (D) NHA cells. Cell survival is presented as percent (%=T/C x 100). Data is given as mean ± SD (n = 3). Error bars represent SD.

## Abbreviations

The following abbreviations are used in this manuscript:

GB	Glioblastoma
TMZ	Temozolomide
MGMT	O6-methylguanine methyltransferase
TAX	Tamoxifen
PEG	Polyethylene glycol
Lf	Lactoferrin
PLGA	Poly(lactic-co-glycolic acid)
NPs	Nanoparticles
PDI	Polydispersity index
EE	Encapsulation efficiency
WHO	World Health Organization
FDA	Food and Drug Administration
PKC	Protein kinase C
CNS	Central nervous system
BBB	Blood–brain barrier
DOE	Design of experiments
PVA	Polyvinyl alcohol
EDC	1-ethyl-3-(3-dimethylaminopropyl) cardodiimide
PBS	Phosphate-buffered saline
DMSO	Dimethyl sulfoxide
TCA	Trichloroacetic acid
SRB	Sulforhodamine B
NaOH	Sodium hydroxide
EA	Ethyl acetate
DMEM	Dulbecco’s Modified Eagle Medium
FBS	Fetal bovine serum
NHA	Healthy human astrocytes
BCA	Bicinchoninic acid
DLS	Dynamic laser scattering
LDV	Laser Doppler velocimetry
LC	Loading capacity
ATR	Attenuated total reflectance
FTIR	Fourier Transform Infrared Spectroscopy
TEM	Transmission electron microscopy
C6	Coumarin-6
SD	Standard deviation
RCs	Regression coefficients
CI	Confidence intervals
EPR	Enhanced permeability and retention
CE	Conjugation efficiency
Tf	Transferrin
