# Supplementary material for: Lactoferrin-Decorated PLGA Nanoparticles for Targeted Tamoxifen Repurposing in Glioblastoma Cells
Source: Polymers (Basel). 2026 Apr 27;18(9):1055. doi: 10.3390/polym18091055 (PMC13165678; doi:10.3390/polym18091055)
Supplement: Supplementary file 1 [file polymers-18-01055-s001.zip › polymers-4245194-supplementary.pdf]

Supplementary Information

1. Central Composite Design

Table S1. Obtained responses for the 35 runs of the DOE, associated to each independent variable combination.

Run order	A	B	C	D	Average Size	PDI	Zeta Potential (mV)	EE (%)	LC (%)
**1**	+1	+1	-1	+1	95.17	0.092	4.53	15.82	1.582
**2**	0	0	0	0	134.6	0.114	1.33	22.07	0.74
**3**	-1	-1	-1	-1	184.3	0.128	-0.668	15.23	1.52
**4**	+1	-1	+1	+1	143.5	0.129	0.0698	61.75	1.24
**5**	0	0	0	0	138.9	0.053	1.92	36.51	1.22
**6**	+1	+1	-1	-1	130.0	0.335	2.09	21.49	2.15
**7**	-1	-1	+1	-1	202.8	0.174	-3.28	55.35	1.107
**8**	0	0	0	0	128.9	0.101	0.338	14.29	0.48
**9**	+1	+1	+1	+1	126.2	0.086	-3.48	27.44	0.549
**10**	-1	+1	+1	+1	146.5	0.141	-2.76	4.689	0.0937
**11**	-1	+1	-1	-1	161.9	0.109	-0.586	4.692	0.4692
**12**	-1	-1	+1	+1	188.7	0.109	0.0717	81.42	1.628
**13**	-1	-1	+1	-1	178.8	0.097	-2.42	62.32	1.246
**14**	-1	+1	+1	-1	162.0	0.113	-3.66	37.7	0.754
**15**	+1	+1	+1	-1	117.0	0.078	-4.53	16.9	0.338
**16**	+1	-1	-1	+1	113.3	0.063	2.92	38.5	3.85
**17**	-1	-1	+1	+1	214.9	0.150	-0.82	84.42	1.688
**18**	+1	+1	-1	-1	114.5	0.350	-4.53	25.65	2.565
**19**	+1	-1	-1	-1	120.1	0.160	0.370	12.83	1.28
**20**	-1	+1	-1	-1	158.5	0.184	0.242	1.44	0.144
**21**	+1	+1	+1	+1	138.2	0.113	-2.63	31.89	0.638
**22**	+1	-1	-1	+1	118.0	0.120	7.84	16.79	1.68
**23**	-1	-1	-1	+1	133.9	0.110	5.35	17.27	1.727
**24**	-1	+1	+1	-1	170.9	0.096	-7.59	49.35	0.987
**25**	+1	-1	+1	-1	163.5	0.227	-2.85	10.09	0.2017
**26**	+1	-1	+1	+1	176.1	0.092	0.283	81.54	1.631
**27**	+1	-1	+1	-1	175.3	0.310	-2.3	9.44	0.1888
**28**	+1	+1	-1	+1	100.6	0.153	1.54	25.57	2.56
**29**	-1	-1	-1	-1	153.7	0.075	5.43	10.57	1.057
**30**	-1	+1	-1	+1	154.0	0.146	1.06	22.19	2.219
**31**	+1	+1	+1	-1	123.6	0.051	-7.26	15.68	0.3135
**32**	-1	+1	-1	+1	180.6	0.136	1.54	59.97	5.997
**33**	+1	-1	-1	-1	110.6	0.054	7.16	17.68	1.768
**34**	-1	+1	+1	+1	136.6	0.082	-2.55	36.77	0.745
**35**	-1	-1	-1	+1	125.4	0.090	4.94	17.49	1.749

Table S2. Results of the ANOVA statistical analysis of the DOE for response 1, Size.

Response 1: Size					
	**SS**	**dF**	**MS**	**F-value**	**p-value**
**Model**	26608.32	15	1773.89	11.2	< 0.0001
**A-Number of Sonication Cycles**	10798.25	1	10798.25	68.21	< 0.0001
**B-PVA Concentration**	2567.4	1	2567.4	16.22	0.0007
**C-Amount of PLGA**	5253.89	1	5253.89	33.19	< 0.0001
**D-EA/PVA Ratio**	576.56	1	576.56	3.64	0.0716
**AB**	126.52	1	126.52	0.7992	0.3825
**AC**	393.61	1	393.61	2.49	0.1313
**AD**	74.33	1	74.33	0.4695	0.5015
**BC**	4017.89	1	4017.89	25.38	< 0.0001
**BD**	6.82	1	6.82	0.0431	0.8378
**CD**	249.93	1	249.93	1.58	0.2242
**ABC**	283.4	1	283.4	1.79	0.1967
**ABD**	23.51	1	23.51	0.1485	0.7042
**ACD**	8.03	1	8.03	0.0507	0.8242
**BCD**	163.67	1	163.67	1.03	0.322
**ABCD**	2064.51	1	2064.51	13.04	0.0019
**Residual**	3008	19	158.32		
**Lack of Fit**	488.16	1	488.16	3.49	0.0782
**Pure Error**	2519.84	18	139.99		
**Cor Total**	29616.32	34			
**Fit Statistics**			**R^2**	**Adjusted R^2**	**Predicted R^2**
			0.9683	0.9482	0.9413

Table S3. Results of the ANOVA statistical analysis of the DOE for response 2, PDI.

Response 2: PDI					
	SS	dF	MS	F-value	p-value
**Model**	0.1491	15	0.0099	5.85	0.0002
**A-Number of Sonication Cycles**	0.007	1	0.007	4.12	0.0567
**B-PVA Concentration**	0.001	1	0.001	0.5763	0.4571
**C-Amount of PLGA**	0.0021	1	0.0021	1.22	0.2841
**D-EA/PVA Ratio**	0.0166	1	0.0166	9.78	0.0056
**AB**	0	1	0	0.0155	0.9023
**AC**	0.0016	1	0.0016	0.9313	0.3467
**AD**	0.0155	1	0.0155	9.14	0.007
**BC**	0.0475	1	0.0475	27.97	< 0.0001
**BD**	7.81E-07	1	7.81E-07	0.0005	0.9831
**CD**	0.0018	1	0.0018	1.07	0.3143
**ABC**	0.015	1	0.015	8.83	0.0078
**ABD**	0.0001	1	0.0001	0.0309	0.8623
**ACD**	0.0014	1	0.0014	0.8035	0.3813
**BCD**	0.0215	1	0.0215	12.64	0.0021
**ABCD**	0.0181	1	0.0181	10.65	0.0041
**Residual**	0.0323	19	0.0017		
**Lack of Fit**	0.006	1	0.006	4.09	0.0581
**Pure Error**	0.0263	18	0.0015		
**Cor Total**	0.1814	34			
**Fit Statistics**			**R^2**	**Adjusted R^2**	**Predicted R^2**
			0.9083	0.8500	0.8389

Table S4. Results of the ANOVA statistical analysis of the DOE for response 3, Zeta Potential.

**Response 3: Zeta Potential**					
	**SS**	**dF**	**MS**	**F-value**	**p-value**
**Model**	369.44	15	24.63	4.68	0.001
**A-Number of Sonication Cycles**	0.7574	1	0.7574	0.1438	0.7088
**B-PVA Concentration**	80.23	1	80.23	15.23	0.001
**C-Amount of PLGA**	225.43	1	225.43	42.79	< 0.0001
**D-EA/PVA Ratio**	55.88	1	55.88	10.61	0.0041
**AB**	0.7366	1	0.7366	0.1398	0.7126
**AC**	0.5781	1	0.5781	0.1097	0.7441
**AD**	0.3958	1	0.3958	0.0751	0.7869
**BC**	0.5622	1	0.5622	0.1067	0.7475
**BD**	0.466	1	0.466	0.0885	0.7694
**CD**	0.1084	1	0.1084	0.0206	0.8874
**ABC**	0.0397	1	0.0397	0.0075	0.9317
**ABD**	1.55	1	1.55	0.2951	0.5933
**ACD**	0.2769	1	0.2769	0.0526	0.8211
**BCD**	0.0732	1	0.0732	0.0139	0.9074
**ABCD**	2.35	1	2.35	0.4464	0.5121
**Residual**	100.09	19	5.27		
**Lack of Fit**	5.36	1	5.36	1.02	0.3261
**Pure Error**	94.72	18	5.26		
**Cor Total**	469.53	34			
**Fit Statistics**			**R^2**	**Adjusted R^2**	**Predicted R^2**
			0.9259	0.8673	0.8013

Table S5. Results of the ANOVA statistical analysis of the DOE for response 4, EE.

Response 4: EE					
	**SS**	**dF**	**MS**	**F-value**	**p-value**
**Model**	15738.92	15	1049.26	8.95	< 0.0001
**A-Number of Sonication Cycles**	542.94	1	542.94	4.63	0.0444
**B-PVA Concentration**	1193.76	1	1193.76	10.19	0.0048
**C-Amount of PLGA**	3688.7	1	3688.7	31.47	< 0.0001
**D-EA/PVA Ratio**	2065.75	1	2065.75	17.63	0.0005
**AB**	109.11	1	109.11	0.931	0.3467
**AC**	1043.87	1	1043.87	8.91	0.0076
**AD**	209.99	1	209.99	1.79	0.1965
**BC**	2053.97	1	2053.97	17.53	0.0005
**BD**	743.37	1	743.37	6.34	0.0209
**CD**	75.25	1	75.25	0.6421	0.4329
**ABC**	371.92	1	371.92	3.17	0.0908
**ABD**	317.82	1	317.82	2.71	0.116
**ACD**	1429.64	1	1429.64	12.2	0.0024
**BCD**	1614.67	1	1614.67	13.78	0.0015
**ABCD**	278.18	1	278.18	2.37	0.1399
**Residual**	2226.75	19	117.2		
**Lack of Fit**	121.13	1	121.13	1.04	0.3224
**Pure Error**	2105.62	18	116.98		
**Cor Total**	17965.67	34			
**Fit Statistics**			**R^2**	**Adjusted R^2**	**Predicted R^2**
			0.9379	0.8889	0.8832

Table S6. Results of the ANOVA statistical analysis of the DOE for response 5, LC.

Response 5: LC					
	**SS**	**dF**	**MS**	**F-value**	**p-value**
**Model**	33.01	15	2.2	3.49	0.0058
**A-Number of Sonication Cycles**	0.0111	1	0.0111	0.0176	0.8959
**B-PVA Concentration**	0.0663	1	0.0663	0.1052	0.7492
**C-Amount of PLGA**	11.24	1	11.24	17.83	0.0005
**D-EA/PVA Ratio**	5.68	1	5.68	9.01	0.0073
**AB**	0.0216	1	0.0216	0.0342	0.8552
**AC**	1.02	1	1.02	1.61	0.2197
**AD**	0.4135	1	0.4135	0.6557	0.4281
**BC**	1.79	1	1.79	2.84	0.1084
**BD**	0.0008	1	0.0008	0.0013	0.9717
**CD**	1.68	1	1.68	2.67	0.119
**ABC**	0.5417	1	0.5417	0.859	0.3656
**ABD**	3.02	1	3.02	4.8	0.0412
**ACD**	2.85	1	2.85	4.52	0.0469
**BCD**	1.74	1	1.74	2.76	0.1132
**ABCD**	2.93	1	2.93	4.64	0.0442
**Residual**	11.98	19	0.6306		
**Lack of Fit**	1.03	1	1.03	1.7	0.2089
**Pure Error**	10.95	18	0.6082		
**Cor Total**	44.99	34			
**Fit Statistics**			**R^2**	**Adjusted R^2**	**Predicted R^2**
			0.9520	0.9215	0.9189

Eq. S1.

$$\text{Size}=146.33-18.37A-8.96B+12.81C-4.24D-1.99AB+3.51AC+1.52AD-11.21BC+0.46BD+2.79CD+2.98ABC-0.86ABD+0.50ACD-2.26BCD+8.03ABCD$$

Eq. S2.

$$\text{PDI}=0.1320+0.0148A+0.0055B-0.0080C-0.0228D+0.0009AB-0.0070AC-0.0220AD-0.0385BC-0.0002BD+0.0075CD-0.0217ABC-0.0013ABD+0.0065ACD+0.0259BCD+0.0238ABC$$

Eq. S3.

$$\text{Zeta Potential}=-0.083+0.154A-1.583B-2.654C+1.321D-0.152AB-0.134AC+0.111AD+0.133BC+0.121BD+0.058CD-0.035ABC+0.220ABD-0.093ACD-0.048BCD-0.271ABCD$$

Eq. S4.

$$\text{EE}=30.37-4.12A-6.11B+10.74C+8.03D+1.85AB-5.71AC+2.56AD-8.01BC-4.82BD+1.53CD+3.41ABC-3.15ABD+6.68ACD-7.10BCD+2.95ABCD$$

Eq. S5.

$$\text{LC}=1.374-0.0186A-0.0455B-0.5928C+0.4215D-0.0260AB-0.1782AC-0.1137AD-0.2365BC-0.0050BD-0.2292CD+0.1301ABC-0.3075ABD+0.2984ACD-0.2331BCD+0.3025ABCD$$

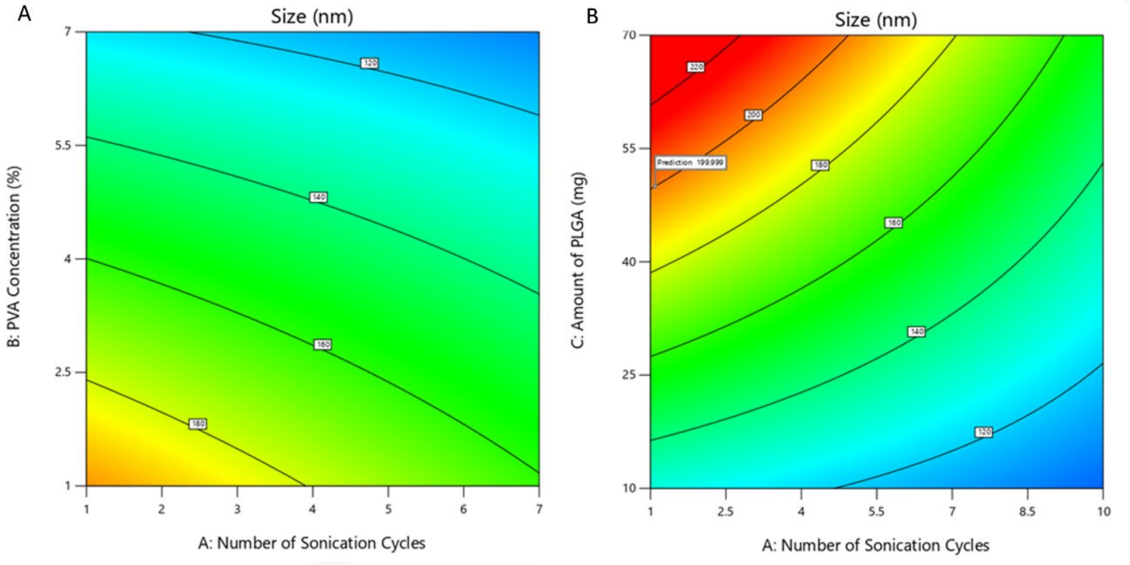

Figure S1. 2D contour plots illustrating the influence of selected formulation and process variables on the particle size of the PLGA NPs. The color gradient ranges from blue to red, indicating smaller to larger sizes, respectively. S1.A- Effect of the number of sonication cycles (A) and PVA concentration (B). S1.B- Effect of the number of sonication cycles (A) and amount of PLGA (C).

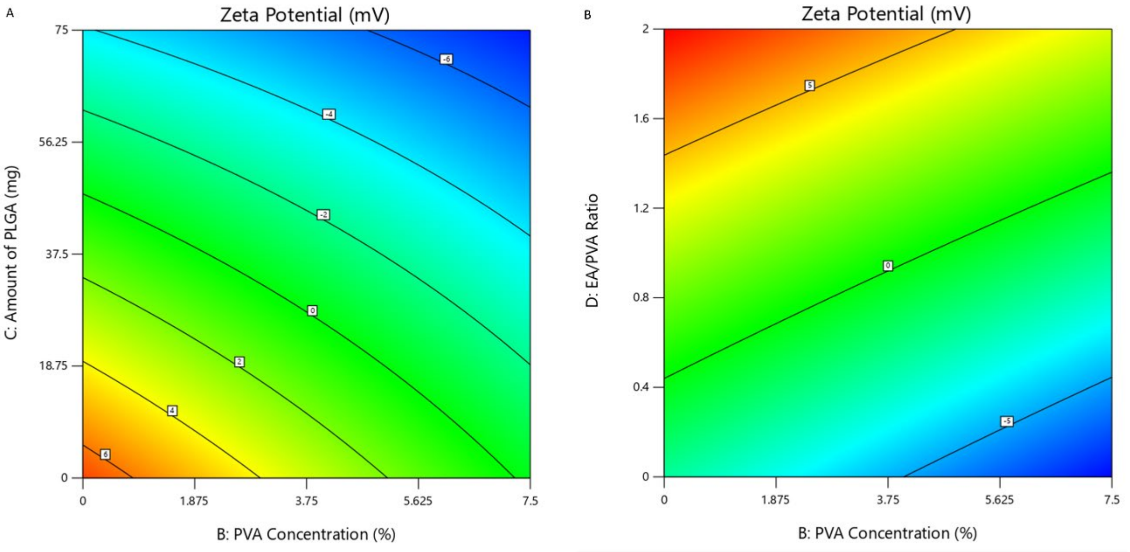

Figure S2. 2D contour plots illustrating the influence of selected formulation and process variables on the zeta potential of the PLGA NPs. The color gradient ranges from blue to red, indicating lower (more negative) to higher (less negative or positive) zeta potential values, respectively. S2.A - Effect of the PVA concentration (B) and amount of PLGA (C). S2.B - Effect of the PVA concentration (B) and EA/PVA ratio (D).

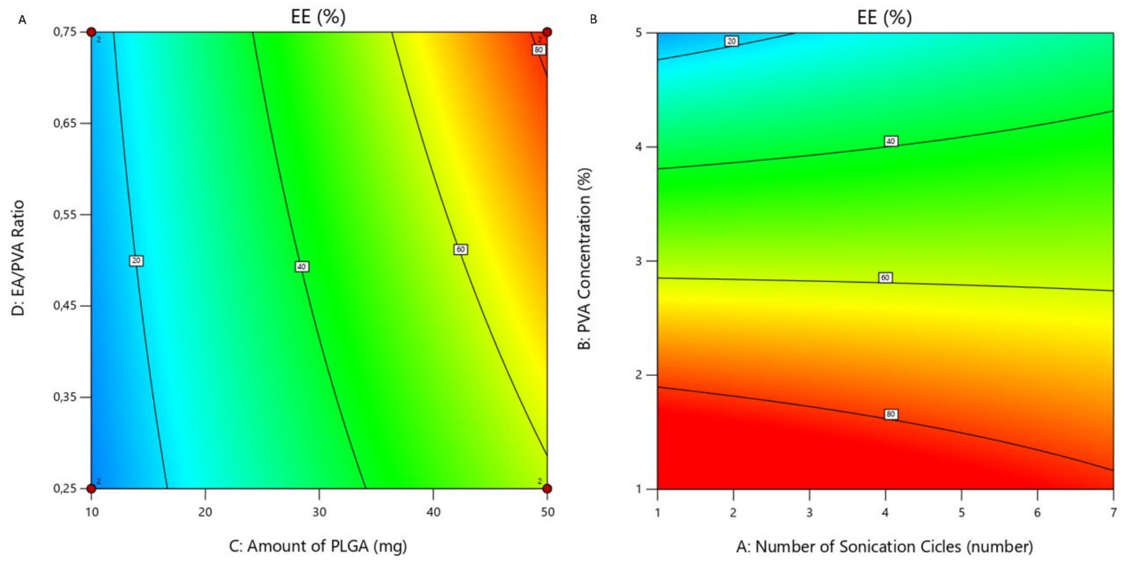

Figure S3. 2D contour plots illustrating the influence of selected formulation and process variables on the EE of the PLGA NPs. The color gradient ranges from blue to red, indicating lower to higher EE values, respectively. S3.A - Effect of the amount of PLGA (C) and the EA/PVA ratio (D). S3.B - Effect of the number of sonication cycles (A) and the PVA concentration (B).

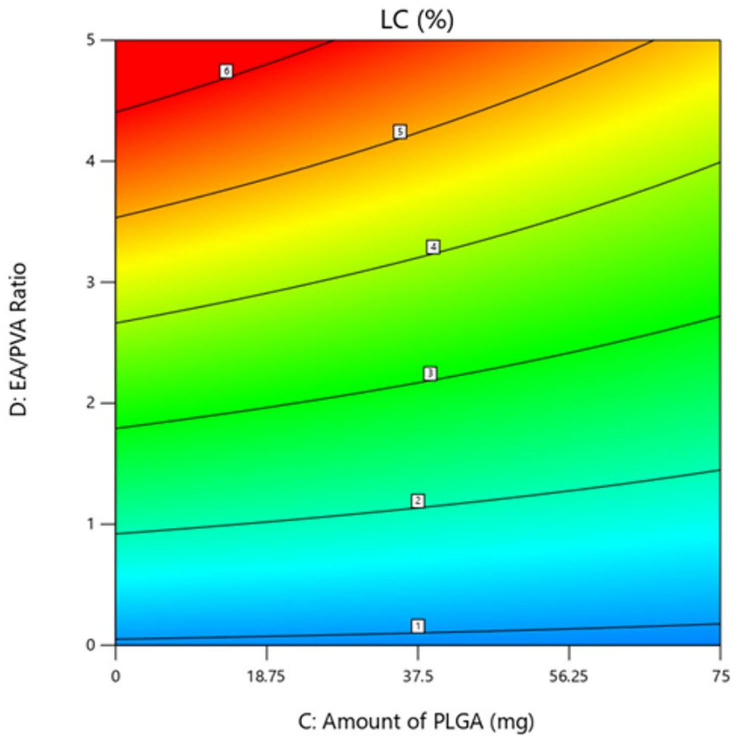

Figure S4. 2D contour plot illustrating the influence of formulation and process variables on the LC of the PLGA NPs. The color gradient ranges from blue to red, representing lower to higher LC values, respectively. The plot displays the combined effect of the amount of PLGA (C) and the EA/PVA volume ratio (D).

2. ATR-FTIR analysis of PEG–PLGA conjugation

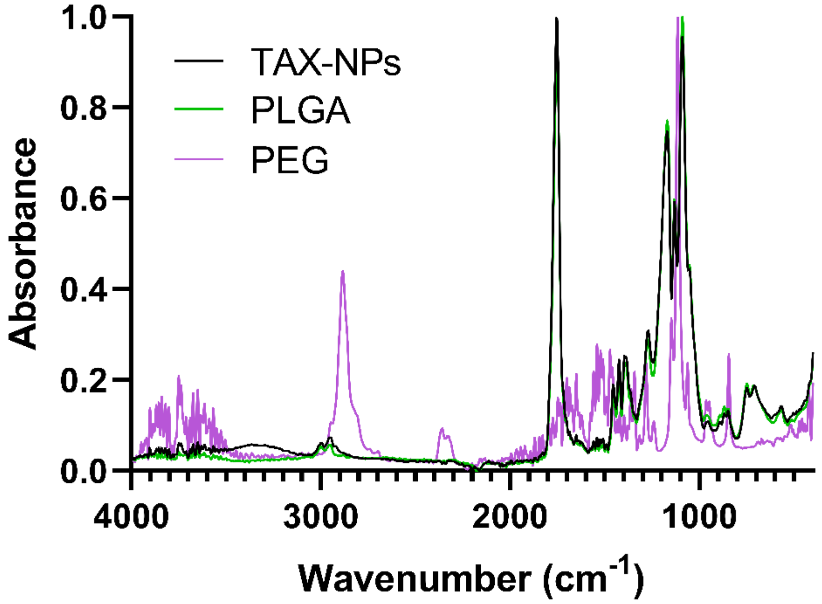

Figure S5. ATR-FTIR absorbance spectra of pegylated TAX-loaded PLGA NPs, pure PLGA, pure PEG and pure TAX.

The FTIR spectra of TAX-loaded NPs exhibited the characteristic vibrational modes of PLGA. Specifically, the ester carbonyl (C=O) stretching vibration appeared prominently at 1759 cm^{-1}, confirming the integrity of the polymer's ester functionality. Additional bands were observed at 1452 cm^{-1}, attributed to CH_2 bending vibrations, and at 1183 cm^{-1} and 1087 cm^{-1}, corresponding to C–O–C asymmetric and symmetric stretching, respectively. The similarity between the spectra of pure PLGA and NPs confirmed that the polymer remained chemically stable during NP formulation. Furthermore, the spectral profile was dominated by PLGA signals, which is consistent with its high proportion relative to PEG. The identification of PEG is particularly challenging not only due to spectral overlap but also due to low PEG:PLGA mass ratio (1:50). Both PLGA and PEG exhibit strong C–O–C stretching bands within the 1000-1300 cm^{-1} region, not allowing direct discrimination of PEG-specific signals in this range in the NPs spectrum. To assess PEG conjugation, attention was also given to the 1500–1700 cm^{-1} region, where weak bands attributed to N–H bending from PEG terminal groups may occur. In this region, subtle but reproducible increases in band intensity were noted in the PEG-modified NPs relative to pure PLGA, suggesting a potential contribution from surface-bound PEG. Furthermore, the ~2880 cm^{-1} band, associated with CH_2 asymmetric stretching commonly found in PEG chains, was not clearly distinguishable from the PLGA background. This overlap limits the conclusions, particularly given that PLGA also contributes CH_2 bands in this range. Overall, although definitive PEG-specific peaks were not isolated, the mentioned minor spectral differences are consistent with low-level PEG presence on the NP surface. Together, the FTIR results support the successful formation of PLGA-based NPs and indicate the partial presence of PEG, although its detection is limited by their low relative abundance and overlapping spectral features.

3. NPs' colloidal stability at storage conditions

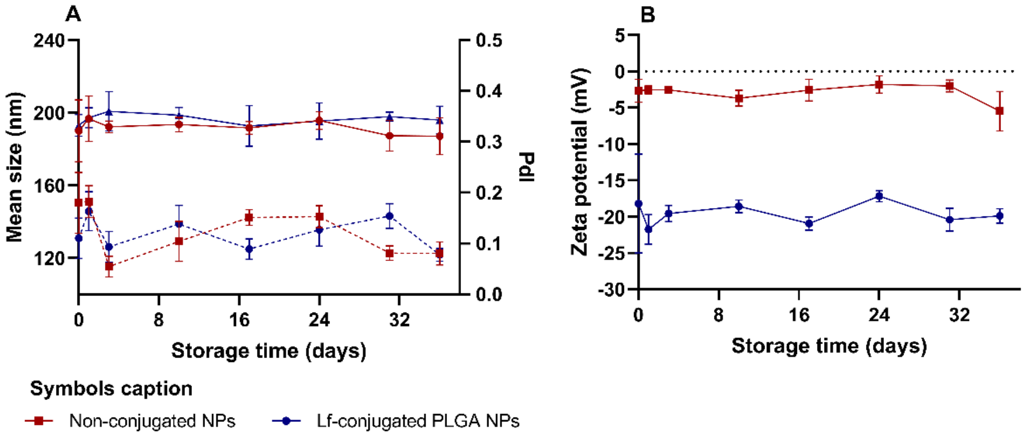

Figure S6. Colloidal stability of Lf-conjugated and non-conjugated TAX-loaded PLGA NPs in storage conditions (4 °C, aqueous suspension) evaluated for 36 days, in terms of changes in (A) mean size (full lines) and PDI values (dotted lines); (B) zeta potential. All data are presented as mean ± SD (n = 3). Error bars represent SD.

4. Cell biocompatibility studies

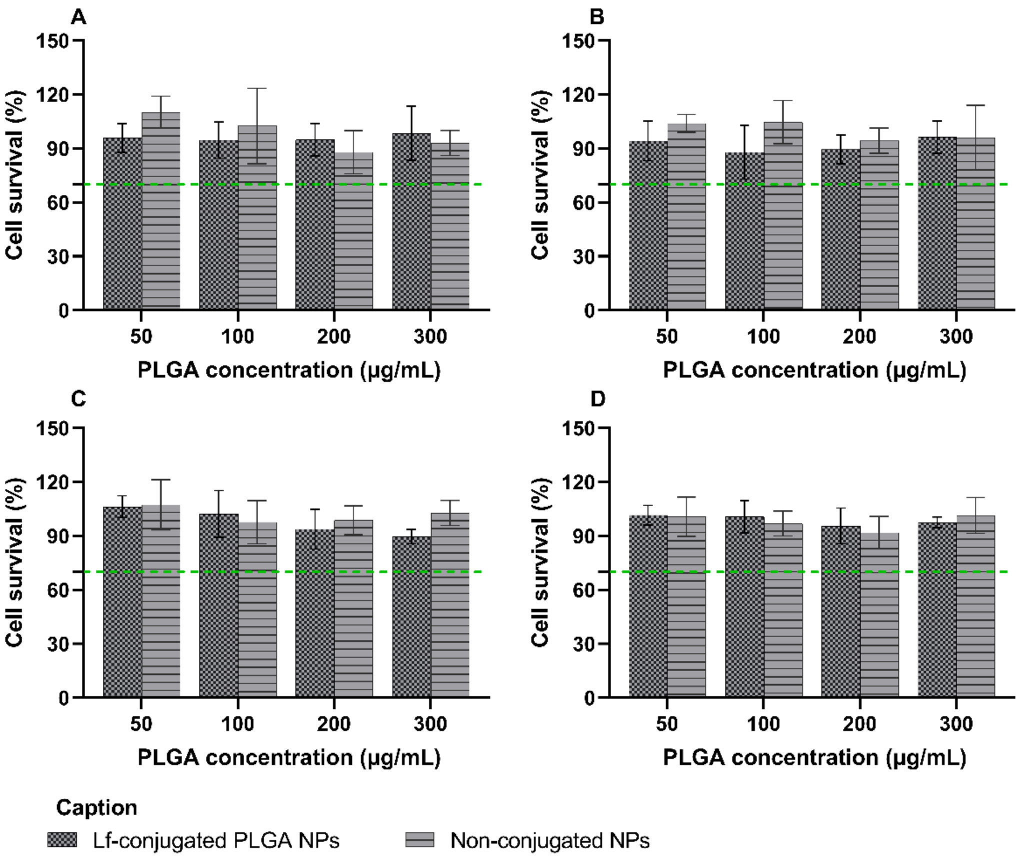

Figure S7. Cell survival quantification by SRB assay after 72 h treatment with control unloaded Lf- conjugated and non-conjugated PLGA NPs on the cell survival of (A) U251, (B) U87, (C) T98G and (D) NHA cells. Cell survival is presented as percent (%=T/C x 100). Data is given as mean ± SD (n = 3). Error bars represent SD.
